# Heat shock proteins IbpA and IbpB are required for NlpI-participated cell division in *Escherichia coli*

**DOI:** 10.3389/fmicb.2015.00051

**Published:** 2015-02-04

**Authors:** Jing Tao, Yu Sang, Qihui Teng, Jinjing Ni, Yi Yang, Stephen Kwok-Wing Tsui, Yu-Feng Yao

**Affiliations:** ^1^Laboratory of Bacterial Pathogenesis, Department of Microbiology and Immunology, Institutes of Medical Sciences, Shanghai Jiao Tong University School of MedicineShanghai, China; ^2^School of Biomedical Sciences, The Chinese University of Hong KongHong Kong, China

**Keywords:** NlpI, *Escherichia coli*, cell division, heat shock protein IbpA and IbpB, stress response

## Abstract

Lipoprotein NlpI of *Escherichia coli* is involved in the cell division, virulence, and bacterial interaction with eukaryotic host cells. To elucidate the functional mechanism of NlpI, we examined how NlpI affects cell division and found that induction of NlpI inhibits nucleoid division and halts cell growth. Consistent with these results, the cell division protein FtsZ failed to localize at the septum but diffused in the cytosol. Elevation of NlpI expression enhanced the transcription and the outer membrane localization of the heat shock protein IbpA and IbpB. Deletion of either *ibpA* or *ibpB* abolished the effects of NlpI induction, which could be restored by complementation. The C-terminus of NlpI is critical for the enhancement in IbpA and IbpB production, and the N-terminus of NlpI is required for the outer membrane localization of NlpI, IbpA, and IbpB. Furthermore, NlpI physically interacts with IbpB. These results indicate that over-expression of NlpI can interrupt the nucleoids division and the assembly of FtsZ at the septum, mediated by IbpA/IbpB, suggesting a role of the NlpI/IbpA/IbpB complex in the cell division.

## Introduction

Bacterial lipoproteins are a group of membrane proteins and play important roles in bacterial physiology and virulence, including nutrient uptake, cell division, antibiotic resistance, cell wall metabolism, transmembrane signal transduction, and adhesion to eukaryotic host cells (Crago and Koronakis, 1998; Egan et al., 2014; Nesta et al., 2014; Zuckert, 2014). Lipoproteins are synthesized as precursor forms harboring an N-terminal signal peptide. In both Gram-negative and Gram-positive bacteria, to form mature lipoprotein, the precursors of lipoproteins are covalently modified by an amide-linked acyl group at the N-terminal cysteine residue and subsequently removed of signal peptide. More than 90 lipoproteins have been annotated in the *Escherichia coli* genome and some of them have been characterized (Ichihara et al., 1981; Yu et al., 1986; Ehlert et al., 1995).

NlpI is a lipoprotein broadly distributed in Gram-negative bacteria and conserved in *E. coli* strains (Ohara et al., 1999). Premature NlpI is a 34-kDa polypeptide containing 294 amino acid residues including an N-terminal signal sequence of 18 amino acid residues. NlpI is located in the outer membrane (OM) and may be processed by Prc protease (Tadokoro et al., 2004; Teng et al., 2010). Moreover, NlpI is a typical Tetratricopeptide repeat (TPR) protein and contains five TPR motifs, which usually mediate intermolecular protein–protein interactions (Das et al., 1998).

NlpI has multiple functions. NlpI contributes to the interaction of *E. coli* with intestine epithelial cells and human brain microvascular endothelial cells (Barnich et al., 2004; Teng et al., 2010). NlpI facilitates the deposition of the complement regulator C4bp to the bacterial surface to evade innate immune system (Tseng et al., 2012). Moreover, the over-production of *nlpI* inhibits the release of bacterial extracellular DNA (eDNA) (Sanchez-Torres et al., 2010). The homolog of NlpI inhibits biofilm formation and contributes to cell cold acclimatization in *Salmonella enterica* serovar Typhimurium (Rouf et al., 2011a,b).

A previous study suggested that NlpI plays a role in the bacterial cell division (Ohara et al., 1999). Insertion inactivation of *E. coli nlpI* results in abnormal cell division and formation of filaments at elevated temperature. Over-expression of *nlpI* in *E. coli* inhibits cell growth and results in the formation of ellipsoids. However, the underlying mechanism of how NlpI regulates cell division remains unknown. The first step in bacterial cytokinesis is the assembly of a stable but dynamic Z ring at the site of division. FtsZ is a tubulin-like filament-forming GTPase and assembles into the Z ring that determines the division plane (Li et al., 2013). The initial placement of FtsZ polymerization site is tightly regulated by multiple mechanisms (Wu and Errington, 2012) as are the subsequent polymer reshaping and force generation that separate the two daughter cells from each other. The interference with FtsZ polymerization disrupts the cell division (Bi and Lutkenhaus, 1993; Mukherjee et al., 1998). It is unclear whether NlpI is associated with FtsZ.

This study aims at understanding the role of NlpI in *E. coli* cell division. We found that the elevation of NlpI protein level not only led to severe inhibition of bacterial growth and the bacterial morphology change, but also inhibited nucleoid division and disturbed FtsZ localization in the septum in *E. coli*. Furthermore, we identified two small heat shock proteins (sHsps), IbpA and IbpB involving in the NlpI-participated cell division regulation and IbpB interacted with NlpI. Our data suggested that NlpI, IbpA, and IbpB form a complex, which most likely plays a role in nucleoid separation and FtsZ localization in cell division.

## Materials and methods

### Bacterial strains and plasmids

The bacterial strains used in this study are derivatives of *E. coli* K12 strain MG1655 or MC1000. All *E. coli* strains and plasmids used in this study are listed in Table 1. All primers used in this study are listed in Table S1.

**Table 1:** **Bacterial strains and plasmids used in this study**.

	**Characteristics**	**Source**
**STRAINS**		
MG1655	*E. coli* K12 strain	Laboratory stock
Δ*nlpI*	The deletion mutant of *nlpI* in MG1655	This study
Δ*ibpA*	The deletion mutant of *ibpA* in MG1655	This study
Δ*ibpB*	The deletion mutant of *ibpB* in MG1655	This study
Δ*ibpAB*	The deletion mutant of *ibpAB* in MG1655	This study
Δ*ompW*	The deletion mutant of *ompW* in MG1655	This study
BTH Reporter	BacterioMatch II Validation Reporter	Stratagene
XL1-Blue MRF'	Host strain for propagating pBT and pTRG recombinants	Stratagene
**PLASMIDS**		
pQE80L	Over-expression vector	Laboratory stock
pQE80-*nlpI*	Over-expression of C-terminal His-tagged protein NlpI	This study
pQE80-*nlpI-*M	Over-expression of C-terminal His-tagged protein NlpI without N-terminal signal sequence	This study
pQE80-*nlpI-*282	Over-expression of truncated NlpI	This study
pQE80-*nlpI-*233	Over-expression of truncated NlpI	This study
pAC-*ibpA*	*ibpA* with its native promoter was cloned into the *EcoR* V and *BamH* I sites of pACYC184	This study
pWM1410	pBAD33-*ftsZ::yfp*	Ma et al., 1996
pRTG	pTRG target plasmid for Bacterial two-hybrid system	Stratagene
pBT	pBT bait plasmid for Bacterial two-hybrid system	Stratagene
pTRG-Gal11P	Bacterial two-hybrid system control plasmid	Stratagene
pBT-LGF2	Bacterial two-hybrid system control plasmid	Stratagene
pBT-*nlpI*	pBT harboring full length of *nlpI*	This study
pBT-*nlpI*-M	pBT harboring *nlpI* without N-terminal signal sequence	This study
pTRG-*ibpA*	pTRG harboring *ibpA*	This study
pTRG-*ibpB*	pTRG harboring *ibpB*	This study

The deletion mutants in *E. coli* were constructed as previously described (Datsenko and Wanner, 2000). The Δ*ibpA* isogenic mutant strain was replacement of *ibpA* by the chloramphenicol resistance cassette in the *E. coli* strain MG1655. The Δ*ibpB*, Δ*ibpAB* (deletion of *ibpA* and *ibpB*), and Δ*ompW* were constructed using the same method. *ibpA* with its native promoter was inserted into pACYC184 vector, and the resultant plasmid pAC-*ibpA* was used for complementation assay. His-tagged NlpI or NlpI-M (mature NlpI without signal peptide) was expressed from pQE80-*nlpI* or pQE80-*nlpI*-M, respectively.

### The growth characteristics of various *E. coli* strains

*E. coli* strain MG1655 and its derivative mutants were transformed individually with the recombinant plasmids pQE80-*nlpI* by calcium chloride transformation method. Overnight cultures of the strains were subcultured in 40 ml LB broth (1:100) and incubated at 37°C with agitation until the OD600 was 0.5 as the zero hour reading. Then the cultures were divided into two bottles. One bottle was added with 0.5 mM isopropyl-β-D-thiogalactopyranoside (IPTG) and the other one was not. The two bottles were incubated in 37°C while shaking at 250 rpm for 5 h. OD600 was monitored every hour by biophotometer (Eppendorf).

### Microscopy monitoring of cells and nucleoids

Bacterial morphology was visualized by light microscopy of Gram-stained cells and scanning electron microscopy. Nucleoids were stained with 4′,6-diamidino-2-phenylindole (DAPI) and observed by an Olympus fluorescence microscope according to the previously described method (Hiraga et al., 1989).

### Microscopy monitoring of FtsZ-YFP

*E. coli* strain MC1000 bearing FtsZ-YFP-expressing plasmid, pWM1410, was transformed with the recombinant plasmid pQE80-*nlpI*, pQE80-*nlpI*-M, pQE80-*nlpI*-282 or pQE80-*nlpI*-233, respectively. Overnight cultures of the strains were subcultured in 40 ml LB broth (1:100), supplemented with ampicillin and chloramphenicol, and incubated at 37°C with agitation until the OD600 was 0.5. Then the cultures were divided into two bottles. One bottle was added with 0.5 mM IPTG and 10mM L-arabinose (Ara), the other one was added with 10 mM Ara. The two bottles were incubated in 37°C while shaking at 250 rpm for 4 h. Bacteria were stained with DAPI. Nucleoids and FtsZ-YFP were observed by fluorescence microscope (Olympus).

### Cell fractionation

Cell fractionation was carried out as described previously (Wai et al., 2003; Zhou et al., 2012). Briefly, the bacterial cells were harvested by centrifugation and washed with 10 mM Tris-HCl buffer (pH 8.0) followed by sonication to disrupt the cells. The cell debris and unbroken cells were removed by centrifugation at 5000 *g* for 10 min at 4°C, and the supernatant was fractionated into the membrane and cytoplasmic fractions by centrifugation at 10,000 *g* for 30 min at 4°C. The supernatant was cytoplasmic fraction (CP). The sediment was treated with N-lauryl sarcosine at a final concentration of 2% at room temperature for 30 min and then centrifuged at 15,000 *g* for 30 min at 4°C. The resulting sediment was OM fraction, and the supernatant was inner membrane fraction. Samples were analyzed by 15% SDS-PAGE. The discrepant bands on the 15% SDS-PAGE were applied to mass spectrometry (MS) analysis by using ABI 4700 TOF/TOF.

### RNA manipulation and quantitative real-time RT-PCR

Total bacterial RNA was extracted using RiboPure-Bacteria kit (Ambion) and treated with DNase I to remove genomic DNA according to the manufacturer's instructions. RNA concentrations were measured using a NanoDrop spectrophotometer (Thermo). Reverse transcription (RT) was implemented using the SuperScript III First-Stand Synthesis System (Invitrogen). The quantification of the target gene mRNA level was performed by the quantitative real-time PCR (qRT-PCR) with a SYBR Premix Ex Taq II (TaKaRa) and the ABI PRISM 7500 Fast Real-Time PCR System according to the manufacturer's instructions. The primers of *nlpI*, *ibpA*, *ibpB*, *ompW* or 16S rRNA (internal control) were listed in Table S1.

### Microarray analysis

Microarray was carried out as described previously (Yao et al., 2005, 2006). A total of 7644 70-mer oligonucleotides from *E. coli* were spotted in replicate onto aminosilane slides. The oligonucleotides that are targeting backbone genes in *E. coli* genomes were derived from an oligonucleotide set (http://pfgrc.jcvi.org/index.php/microarray/array_description/escherichia_coli/version1.html). It is a pan *E. coli* genome chip and covers all the ORFs in *E. coli* strain MG1655. *E. coli* strain MG1655 harboring plasmids pQE80-*nlpI* or pQE80L were grown in LB medium at 37°C with agitation until the OD600 was 0.5. Then IPTG was added at a final concentration of 0.5 mM followed by incubation with shaking for 2 h at 250 rpm. Total RNA was immediately isolated by using RiboPure-Bacteria kit (Ambion) and treated with DNase I. Ten micrograms of total RNA was denatured in the presence of 600 ng of random hexamers and 2 μl of 10X dNTPs [dATP, dCTP, dGTP, and aminoallyl-dUTP (100 mM each)] (total, 20 μl) for 5 min at 65°C and was snap-cooled on the ice. Then, cDNA synthesis was implemented by the SuperScript III First-Stand Synthesis System for RT-PCR kit (Invitrogen). Residual RNA was hydrolyzed by alkaline, and bacterial cDNA was purified by QIAquick PCR Purification Kit (Qiagen) and labeled by use of ARES Alexa Fluor dye at 488 or 594 nm (Molecular Probes, Invitrogen) according to the manufacturer's instructions. The labeled cDNA was purified by Centri-Spin 20 Columns (Princeton Separations). The microarray slides were prehybridized. Equal amounts of two oppositely labeled cDNA were mixed together with equal volume of SlideHybe#3 (Ambion) and loaded onto the slide. The slide was incubated for 16 to 18 h at 42°C and washed at 55°C by 2X SSC/0.1% SDS, 10 min in 0.1X SSC/0.1% SDS at room temperature twice, 5 min in 0.1X SSC at room temperature twice and 2 min in 0.05X SSC. After drying, the slide was scanned at 594 and 488 nm with a GenePix™ 4200A Scanner (Molecular Devices). Three independent experiments were performed by reversing dyes. Image processing and data extraction were accomplished by using GenePix Pro 6.0.1.27 (Axon Instruments). Microarrays were analyzed using R with “limma” package. After loading the microarray data into R, several steps were done following the limma user guides, including: Background Correction (method = “normexp,” offset = 50); Within-Array Normalization (method = “loess”); Between-Array Normalization (method = “quantile”). Two kinds of plots were used to assess the normalization procedures: MA-plot (http://en.wikipedia.org/wiki/MA_plot); Individual-channel Densities.

### Western blot analysis

Protein samples were separated by SDS-PAGE and transferred to PVDF membranes. Membranes were blocked by 5% non-fat milk in Tris Buffered Saline (TBS), with 0.1% Tween-20 added. The rabbit anti-sera against NlpI (1:3000) or anti-IbpA (1:3000) or anti-Crp (1:4000), the mouse anti-sera against-His (1:4000), and the goat horseradish peroxidase (HRP)-conjugated anti-rabbit or anti-mouse IgG antibodies (1: 4000) were used as the primary or secondary antibodies to detect the target proteins. The rabbit anti-sera against NlpI is a gift from Dr. Kwang Sik Kim at Johns Hopkins University. The rabbit anti-IbpA and anti-Crp sera were customized by Beijing ComWin Biotech. The mouse anti-sera against-His was purchased from Tiangen. HRP-conjugated goat anti-rabbit or anti-mouse IgG antibodies were purchased from Beijing ComWin Biotech. The antibody of RNAP (RNA polymerase) was purchased from Santa Cruz and used according to manufacturer's manual. The blots were developed with Super Signal West Pico Chemiluminescent Substrate (Thermo).

### Co-immunoprecipitation (Co-IP) assay

The interactions between NlpI and IbpA or IbpB were analyzed by Co-IP according to the Pierce Crosslink Immunoprecipitation Kit (Thermo) with some modifications. *E. coli* strain MG1655 harboring pQE80-*nlpI*-M were induced with 500 μM IPTG for 4 h and then harvested and resuspended in 1 ml of ice cold buffer (25 mM Tris, 150 mM NaCl, 1 mM EDTA, 1% NP-40, 5% glycerol, pH 7.4). After sonication, cell debris was removed by centrifugation at 10,000 *g* for 30 min at 4°C. Co-IP were performed by binding of 10 μg of anti-HisTag antibody to 20 μl protein A/G plus agarose for 1 h, crosslinking, and then incubating with 150 μg of cell extracts in 300 μl of IP Lysis/Wash buffer for 2 h at 4°C with shaking. The beads were washed with the IP Lysis/Wash buffer. Immune complexes were eluted and analyzed by Western blot using anti-IbpA, anti-NlpI or anti-HisTag antibody.

### Bacterial two-hybrid assay

The protein-protein interaction between NlpI and IbpA or IbpB was examined by BacterioMatch II Two-Hybrid System (Stratagene) according to the previous description (Du et al., 2012). The *nlpI*-M and *nlpI* were cloned into the pBT individually. The *ibpA* and *ibpB* genes were cloned into the pTRG individually. The reporter strain was co-transformed with the recombinant plasmids pBT-*nlpI*-M and pTRG-*ibpA* or pTRG-*ibpB* and streaked onto the dual selective screening medium (DSSM) containing 5 mM 3-amino-1,2,4-triazole (3-AT), 12.5 mM streptomycin, 12.5 mM tetracycline, 25 mM chloramphenicol and 50 mg/ml kanamycin. A cotransformant containing pBT-LGF2 and pTRG-Gal11P was used as a positive control for expected growth on DSSM. A cotransformant containing pBT and pTRG was used as a negative control. Bacterial two-hybrid assay between the recombinant plasmids pBT-*nlpI* and pTRG-*ibpA* or pTRG-*ibpB* were carried out similarly.

### Statistical analysis

qRT-PCR data are shown as means ± standard deviations. Statistical analyses were performed using GraphPad Prism 5. Paired *t*-tests were used to determine *P*-values. With regard to the microarray data analysis, after analyzed by R package, genes with the expression fold change greater than 2 or less than 0.5 and *P*-value < 0.01 were considered as differential expression.

## Results

### Inhibition of *E. coli* growth by induction of *nlpI*

**Figure 1:**
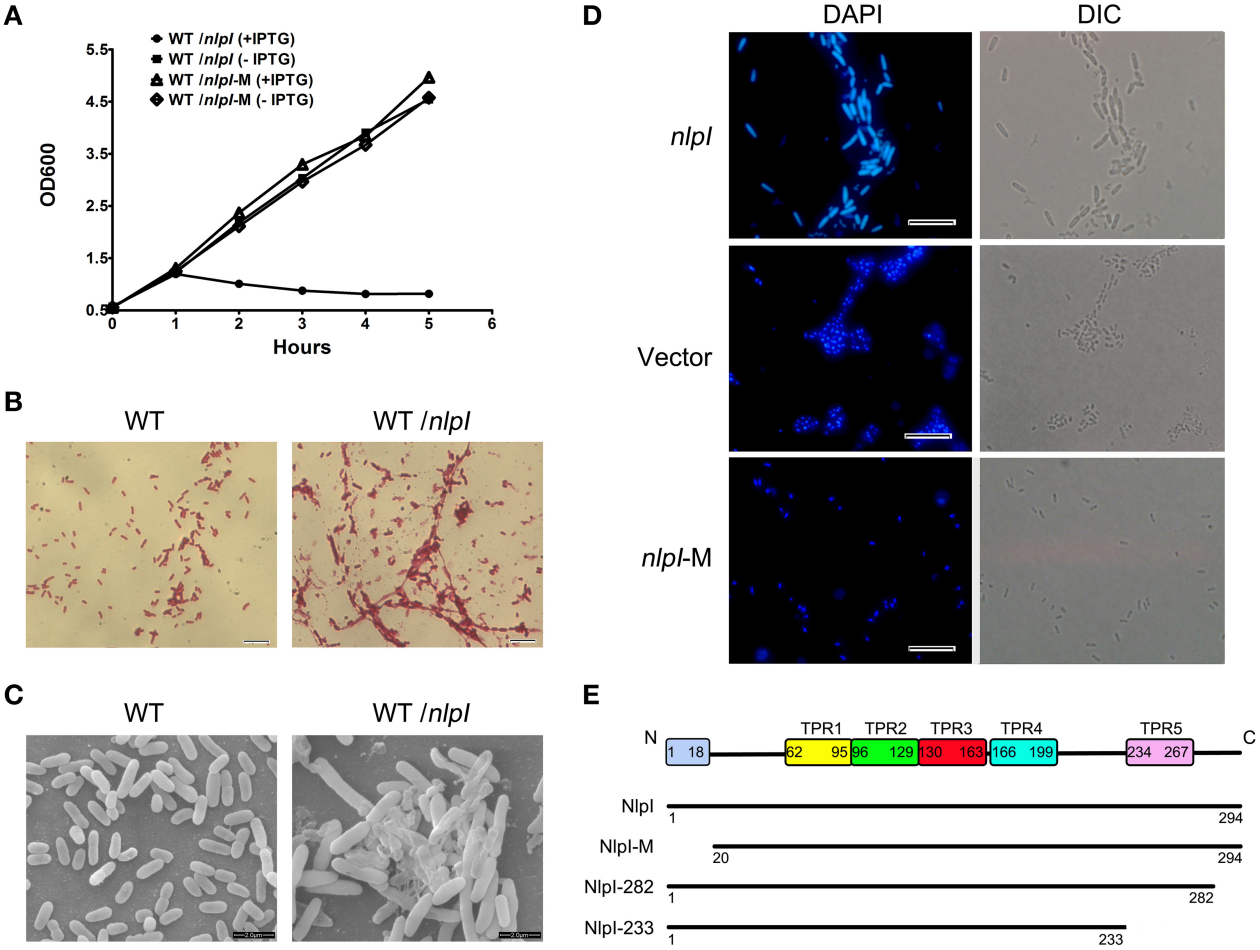
**The elevation of *nlpI* in *E. coli* inhibited the host cell growth. (A)** The growth curves of the wild type (WT) strain MG1655 harboring the plasmid pQE80-*nlpI* or pQE80-*nlpI*-M in the presence or absence of inducer. Results shown are representative of three independent experiments. **(B)** The cellular morphology of Gram-stained bacteria. *E. coli* strain MG1655 harboring plasmid pQE80-*nlpI* was incubated in LB broth at 37°C with shaking until OD600 reached 0.5. Then the bacteria were induced by adding IPTG to a final concentration of 0.5 mM for 4 h. The cells were subsequently stained with Gram stain and observed by light microscope. Magnification, X1000. **(C)** Scanning electron microscopy examined the cellular morphology change and aggregation. Magnification, X10,000. **(D)** Bacteria were stained with DAPI and nucleoids were observed by fluorescence microscopy. Magnification, X1000. Scale bar, 10 μm. **(E)** Schematic presentation of NlpI and its three variants. The full length NlpI contains 294 amino acids including an N-terminal signal sequence and five TPR motifs. NlpI-M represents a mature NlpI protein without N-terminal signal sequence. NlpI-282 and NlpI-233 are truncated NlpI lacking the C-terminal 12 residues and the fifth TPR, respectively.

The previous study found that the over-expression of *nlpI* affects cell growth and cellular morphology in *E. coli* strain MO101 (Ohara et al., 1999). It promoted us to test whether this phenotype is unique in a certain *E. coli* strain or not. Therefore, *E. coli* strain MG1655 harboring plasmid pQE80-*nlpI* was tested. When the bacteria were induced with 0.5 mM IPTG at 37°C, the cell growth was inhibited severely (Figure 1A). Light microscopy showed the increase of width and length of cell and the aggregation of bacterial cells after induction (Figure 1B), and scanning electron microscopy illustrated the appearance of swollen prolate ellipsoids and cell envelope invagination and damage (Figure 1C). Since the bacterial filamentation was observed with over-expression of *nlpI*, we want to check the nucleoids division in these cells. DAPI staining showed that the bacterial nucleoids were anomalous after the induction of *nlpI* compared with control cells (Figure 1D). We suspect that over-expression of *nlpI* affected the cell division by influencing nucleoids division. Another *E. coli* strain DH5α was applied to the above assays and showed similar phenotypes (data not shown). This means that the phenotypes we observed are not strain specific.

During export of OM lipoprotein across the cytoplasmic membrane, the lipoprotein signal peptide is cleaved by signal peptidase, which is critical for the function of lipoprotein (Zuckert, 2014). In order to investigate the role of NlpI signal peptide in bacterial growth and division, we constructed pQE80*-nlpI-*M that contained the sequence in accordance with the mature polypeptide (residues 20–294, lacking the signal sequence and Cys19) (Figure 1E). With the IPTG induction, the growth curve showed the bacterial growth rate was comparable to that of control strain without induction (Figure 1A). DAPI staining showed that the bacterial nucleoids were normal (Figure 1D). This highly suggests that the signal peptide sequence is required for the phenotypes caused by NlpI over-expression.

**Figure 2:**
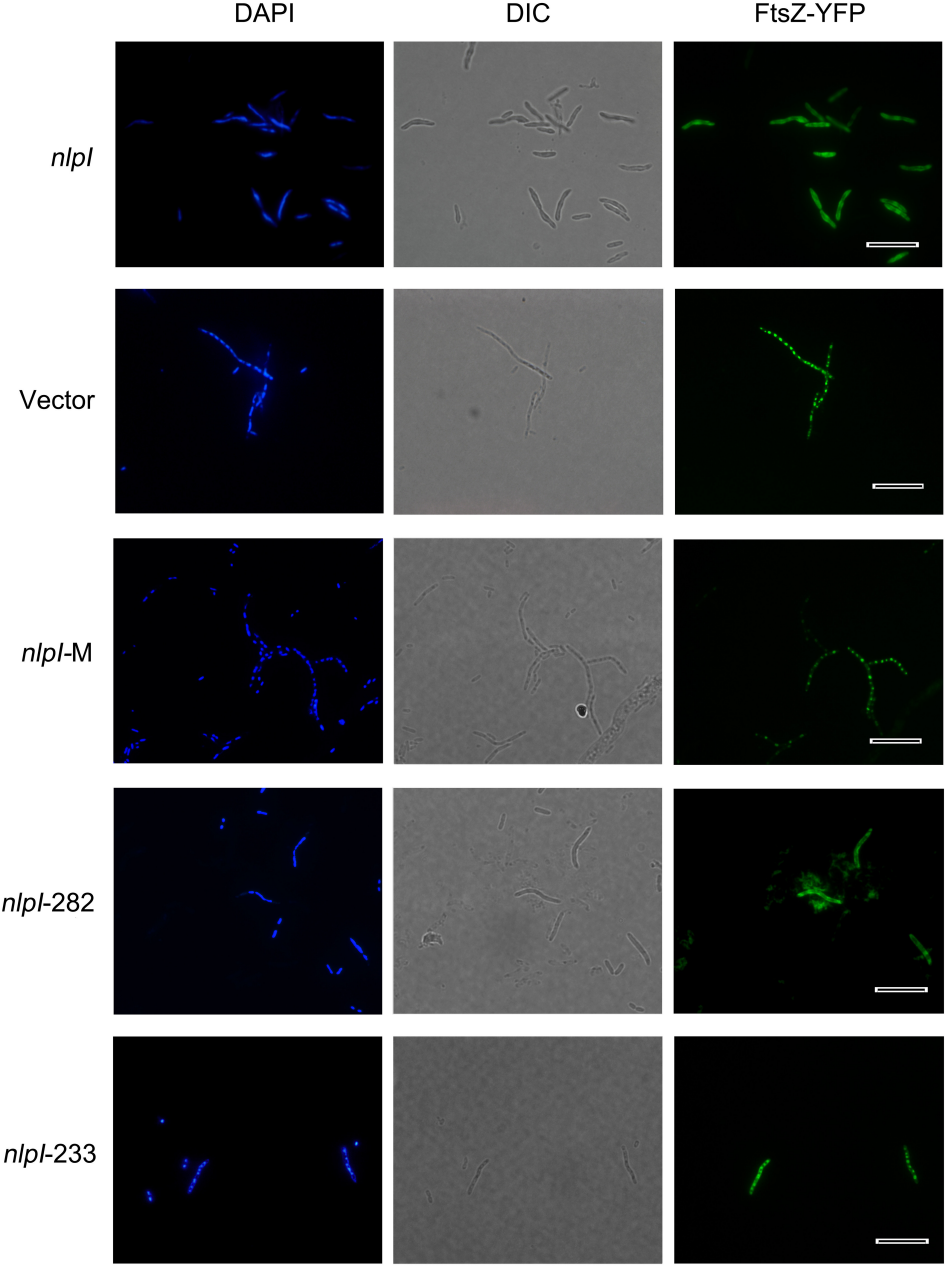
**Elevation of nlpI in *E. coli* disturbed FtsZ localization**. Plasmids pQE80-*nlpI*, pQE80-*nlpI*-M, pQE80-*nlpI*-282 and pQE80-*nlpI*-233 were transformed individually into *E. coli* strain MC1000 carrying *ftsZ-yfp* fusion plasmid pWM1410, the resultant strains were induced with 0.5 mM IPTG and 10 mM Ara for 4 h. Bacteria were stained with DAPI. Nucleoids and FtsZ-YFPs were observed by fluorescence microscope. Magnification, X1000. Scale bar, 10 μm.

It has been shown that the C-terminus of NlpI is critical to correct the thermosensitivity of the *nlpI* mutant (Tadokoro et al., 2004). Moreover, the amino acid sequence showed that NlpI contains five TPR motifs (Wilson et al., 2005). To determine the role of NlpI C-terminus, we constructed two plasmids, pQE80-*nlpI*-282 (lacking the C-terminal 12 residues) and pQE80-*nlpI*-233 (lacking the C-terminal 61 residues including the fifth TPR) (Figure 1E). These two plasmids were introduced individually into *E. coli* strain MG1655 followed by induction with IPTG. We found that the over-expression of *nlpI*-282 severely inhibited the bacterial growth, but *nlpI*-233 failed (data not shown). DAPI staining showed the bacterial nucleoids in the over-expression of *nlpI*-282 cell were anomalous, which was similar to that of the full length *nlpI* (Figure 2), while the nucleoids were normally divided in the *nlpI*-233 over expression cells (Supplementary Figure S1). The above experiments suggested that NlpI was involved in the bacterial division in *E. coli*, and both N-terminal and C-terminal sequences were critical to the role of NlpI in the cell division.

### Elevation of *nlpI* protein level disturbed FtsZ localization

Bacterial cell division requires accurate localization of the cytoskeletal protein FtsZ at the nascent division site and assembly into Z-ring (Bi and Lutkenhaus, 1991). Since over-expression of *nlpI* inhibited nucleoids division in *E. coli*, we speculate that the increase of NlpI protein level perhaps influences FtsZ localization. We induced *nlpI* and *ftsZ-yfp* with IPTG and arabinose, respectively in *E. coli* strain MC1000, and found that the nucleoids were anomalous and FtsZ-YFPs were diffusive in the cytoplasm in *nlpI* over-expression cells. Induction of *nlpI*-282 and *ftsZ-yfp* caused the same change of the nucleoids and FtsZ localization as that of the full length of *nlpI* (Figure 2). However, both nucleoid division and FtsZ localization were normal after induction of *nlpI*-M or *nlpI*-233 (Figure 2). These results indicated that increase of NlpI inhibited nucleoid division and interfered with FtsZ localization, and both N-terminus and C-terminus contributed to this process.

### The transcriptome with elevation of *nlpI* protein level

The above results suggest that NlpI is involved in the nucleoid separation and FtsZ localization. Since we failed to detect the interaction of NlpI and FtsZ in Co-IP assay (data not shown), we performed microarray analyses to identify other factors that may participate in this process. The total RNA from *E. coli* strain MG1655 harboring plasmid pQE80-*nlpI* or pQE80L induced with IPTG were isolated followed by reverse transcription, fluorescent labeling, hybridization, microarray scanning, and data were analyzed as previous description (Yao et al., 2005, 2006).

**Figure 3:**
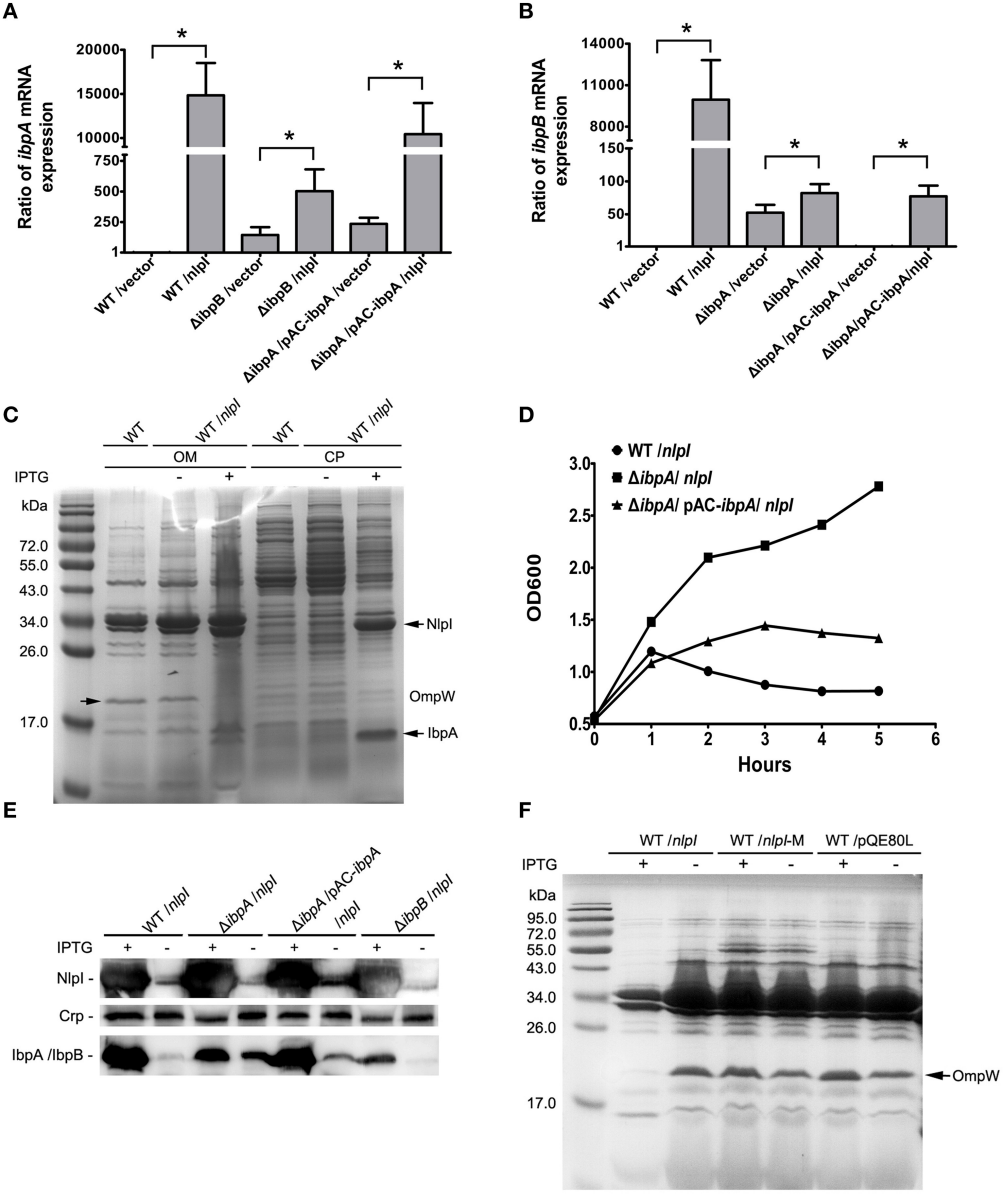
**IbpA and IbpB are required for the NlpI-participated cell division. (A)** The levels of *ibpA* mRNA were significantly increased with induction of *nlpI* in wild type strain or complementation strain. The level of *ibpA* mRNA in wild type strain with vector was set at one. The levels of *ibpA* mRNA in MG1655/pQE80-*nlpI* and Δ*ibpB*/pQE80-*nlpI* were increased 14,839.7 ± 3672.3 and 502.2 ± 178.8 fold, respectively, compared with control. The level of *ibpA* mRNA in Δ*ibpA*/pAC-*ibpA*/pQE80-*nlpI* was increased by 10,437.3 ± 3536.5 fold. The error bars indicated the SD representing the means from three independent experiments. **(B)** The levels of *ibpB* mRNA were the remarkably increased with over-expression of *nlpI* in wild type strain. The level of *ibpB* mRNA of wild type strain with empty vector was set at 1. The levels of *ibpB* mRNA of wild type strain and Δ*ibpA* with inducer were increased by 9947.3 ± 2867.9 and 82.2 ± 13.6 fold, respectively. The level of *ibpB* mRNA in Δ*ibpA*/pAC-*ibpA* was increased by 77.2 ± 16.3 fold. **(C)** The induction of *nlpI* caused the dramatic change of protein profiles of CP and OM of the host cells. Mass spectrometry identified two significantly discrepant bands, IbpA and OmpW. **(D)** The growth curves of wild type strain, Δ*ibpA* and the complementation strain of Δ*ibpA* with over-expression of *nlpI* were determined. The growth of wild type strain and the complementation strain of Δ*ibpA* were inhibited. **(E)** Western blot analysis of the protein levels of NlpI or IbpA/IbpB in CP of the host cells, using polyclonal antibodies anti-NlpI, anti-Crp and anti-IbpA. Crp is a cytoplasmic protein marker. **(F)** The OM protein profiling of MG1655/pQE80-*nlpI*, MG1655/pQE80-*nlpI*-M and MG1655/pQE80L with/without inducer. ^*^P < 0.05.

A total of 142 genes (68 up-regulated and 74 down-regulated genes) were found to be differentially expressed (Supplementary Tables S2 and S3). Some up-regulated and down-regulated genes were listed in Table 2. We did not find the change of transcriptional level of *ftsZ*, but we found that the transcriptional level of genes encoding heat shock proteins and chaperones, such as *ibpA*, *ibpB*, *groES*, *groEL*, *htpG*, *clpB*, *dnaK*, were significantly increased under NlpI overexpression. The expression of genes encoding outer membrane proteins, such as *ompW* and *slp* was significantly decreased. Small heat shock proteins lbpA and lbpB (inclusion body-associated protein) protect heat-denatured proteins from irreversible aggregation (Kuczynska-Wisnik et al., 2004). The heat shock proteins and chaperones including GroEL–GroES, DnaK, and ClpB are involved in preventing aggregation of heat-denatured proteins (Mogk et al., 2002; Sorensen and Mortensen, 2005). We suspect that the significantly up-regulated expression of above genes may be a concomitant event of the elevation of NlpI protein level and play an important role in the NlpI-participated cell division. Quantitative RT-PCR (qRT-PCR) showed that the level of *ibpA* mRNA was increased by 14,839.7 ± 3672.3 fold (means ± standard deviations), and that the levels of *ibpB* mRNA was obviously increased by 9947.3 ± 2867.9 fold in the NlpI over-expression cells (Figures 3A,B), and the transcriptional level of *ompW* was decreased dramatically (Supplementary Figure S2A). These findings confirmed the result of microarray (Table 2), and illustrated that the transcription of *ibpA* and *ibpB* was activated by over-expression of *nlpI*.

**Table 2:** **Genes with differential expression after the elevation of *nlpI***.

**Annotation**	**Gene**	**LogFC**	***P*-value**
Heat shock protein	*ibpA*	7.25	0.00E+00
Heat shock protein	*ibpB*	7.04	0.00E+00
Dihydrolipoamide dehydrogenase	*lpdA*	4.00	0.00E+00
Co-chaperonin	*groES*	3.96	0.00E+00
Heat shock protein 90	*htpG*	3.69	0.00E+00
Chaperonin	*groEL*	3.68	1.00E−02
Cold shock protein	*cspE*	3.44	1.00E−02
D-ribose transporter subunit	*rbsB*	3.23	0.00E+00
DNA-binding transcriptional activator	*marA*	3.05	0.00E+00
Protein disaggregation chaperone	*clpB*	2.57	0.00E+00
Alpha-ketoglutarate transporter	*kgtP*	2.53	0.00E+00
Oligopeptide ABC transporter	*oppA*	2.53	0.00E+00
Molecular chaperone	*dnaK*	2.36	0.00E+00
Galactitol-specific PTS system component IIB	*gatB*	2.35	0.00E+00
ATP-dependent protease ATP-binding subunit	*hslU*	2.32	0.00E+00
Trehalose(maltose)-specific PTS system components IIBC	*treB*	2.14	0.00E+00
Universal stress protein	*uspA*	2.03	0.00E+00
RNA polymerase sigma factor	*rpoS*	2.01	0.00E+00
Biofilm formation regulatory protein	*bssS*	1.94	1.00E−02
ATP-dependent Clp protease proteolytic subunit	*clpP*	1.89	1.00E−02
Heat shock protein	*htpX*	1.88	0.00E+00
Chaperone protein	*dnaJ*	1.66	0.00E+00
Thiamine transporter membrane protein	*thiP*	1.27	0.00E+00
Anti-sigma 28 factor FlgM	*flgM*	−2.05	3.96E−03
Outer membrane protein W	*ompW*	−1.91	9.83E−04
Outer membrane protein	*slp*	−1.80	8.73E−04
Cytochrome d terminal oxidase, polypeptide subunit I	*cydA*	−1.64	1.22E−03
Melibiose:sodium symporter	*melB*	−1.38	5.16E−03
Glutamine ABC transporter periplasmic protein	*glnH*	−1.32	4.36E−03
F0F1 ATP synthase subunit beta	*atpD*	−1.27	3.15E−03
Methyl-accepting chemotaxis protein III	*trg*	−1.23	7.49E−03
Glucose−1-phosphatase/inositol phosphatase	*agp*	−1.19	3.99E−03
Anaerobic glycerol-3-phosphate dehydrogenase subunit B	*glpB*	−1.14	6.57E−03
Citrate reductase cytochrome c-type subunit	*napB*	−1.14	6.80E−03
Ammonium transporter	*amtB*	−1.13	9.61E−03
Chemotaxis regulatory protein CheY	*cheY*	−1.13	6.08E−03
Maltose transporter permease	*malG*	−1.11	5.05E−03
Anaerobic dimethyl sulfoxide reductase subunit B	*dmsB*	−1.06	7.99E−03
Undecaprenyldiphospho-muramoylpentapeptide beta-N-acetylglucosaminyltransferase	*murG*	−1.05	5.45E−03

*LogFC, the log2-fold-change*.

### *ibpA* and *ibpB* are required for the *nlpI*-participated cell division

Since the over expression of *nlpI* cause the expression change of genes, such as *ibpA*, *ibpB* and *ompW*, we speculate that the protein profiling in the *nlpI*-induced cells differs from that of control cells. The bacterial cells were separated into two fractions: CP and OM. SDS-PAGE analysis showed that the over-expression of *nlpI* caused the dramatic changes of the proteins profiling in CP and OM of the host cells compared with those of the control cells (Figure 3C). The two significantly discrepant bands on the SDS-PAGE were applied to MS analysis. One band with increased amount was identified as the heat shock protein IbpA, while the other decreased band was outer membrane protein OmpW (Supplementary Table S4). These findings confirmed the result of microarray and qRT-PCR, and indicated that the elevation of NlpI protein level affected bacterial transcriptome and proteome.

Since the expression of *ibpA* and *ibpB* are significantly elevated in NlpI over expression cells, we sought to unveil their contribution in NlpI-participated cell division. The *ibpA* and *ibpB* form an operon, and are regulated by the σ^32^ protein, which is encoded by *rpoH*. The open reading frames are separated by 111 bp. A σ^54^-dependent promoter locates upstream of *ibpB* (Allen et al., 1992; Kuczynska-Wisnik et al., 2001). IbpA and IbpB appear in aggregated protein fractions after heat shock (Laskowska et al., 1996). To test whether IbpA and IbpB are involved in the NlpI-participated cell division, we constructed the deletion mutants of *ibpA*, *ibpB*, and *ibpAB* (*ibpA* and *ibpB*) in *E. coli*, and induced *nlpI* in these mutants. Surprisingly, the growth curves showed that the elevation of NlpI did not inhibit cell growth of these three deletion mutants (Figure 3D, Supplementary Figure S2B). After complementation of a copy of *ibpA* with its native promoter into the deletion mutant of *ibpA*, we found that over-expression of *nlpI* could inhibit the cell growth (Figure 3D). qRT-PCR showed that the level of *ibpA* mRNA in the complementation strain was restored to the level of the wild type strain (Figure 3A). The inhibition of cell growth by the over-expression of *nlpI* was abolished when *ibpB* or *ibpAB* was deleted (Supplementary Figure S2B). Interestingly, qRT-PCR results showed that the level of *ibpA* mRNA in Δ*ibpB* was increased by 502.2 ± 178.8 fold after the *nlpI* induction, but was significantly lower than in MG1655 or the complementation strains of Δ*ibpA* (Figure 3A, Supplementary Figure S2C). Likewise, we detected that the level of *ibpB* mRNA in Δ*ibpA* was increased by 82.2 ± 13.6 fold after the *nlpI* over-expression, which was strikingly lower than that of the wild type strain with IPTG induction (Figure 3B). These results indicated that both IbpA and IbpB were required for the NlpI-participated cell division, and IbpA promoted the transcription level of *ibpB* in the over-expression of *nlpI* cells, and *vice versa*.

qRT-PCR showed that the transcriptional level of *ibpB* was increased by 52.4 ± 11.9 fold in Δ*ibpA* cells compared with wild type strain, which could be restored by trans-complementation (Figure 3B). We prepared the polyclonal antibody of IbpA and NlpI and tested the expression of NlpI and IbpA/IbpB in the cytoplasm by Western blot. Since IbpA and IbpB, whose molecular masses were both about 16-kDa, shared 48% identity at the amino acid level, the IbpA polyclonal antibody can recognize both IbpA and IbpB. We found the result of Western blot was consistent with that of qRT-PCR (Figure 3E). Western blot detected IbpB in Δ*ibpA* cells and IbpA in the complementation strain of Δ*ibpA* (Figure 3E). These results showed that IbpA inhibited transcription of *ibpB* in wild type strain in accord with the previous finding (Gaubig et al., 2011). The level of *ibpA* mRNA were increased by 144.2 ± 64.4 fold in Δ*ibpB* cells compared with that of wild type strain (Figure 3A). This means that IbpB inhibited the transcription of *ibpA*.

With the elevation of NlpI, DAPI staining showed that the nucleoids divided normally in both Δ*ibpA* and Δ*ibpB*, but the nucleoids were anomalous in the complementation strains of Δ*ibpA* (Supplementary Figure S2D). Furthermore, the wild type strain MG1655 with either over-expression of *ibpA* or *ibpB*, or both *ibpA* and *ibpB*, showed normal growth and nucleoids division (Supplementary Figure S2D). Therefore, these data indicated that nucleoids division defect cause by NlpI overexpression was dependent on IbpA and IbpB in *E. coli*, and other factor(s) may be involved in this process.

To further test whether the decrease of OmpW is related to the NlpI-participated cell division, we constructed the deletion mutant of *ompW* followed by the over-expression of *nlpI*. The growth of Δ*ompW* was inhibited as the wild type strain when *nlpI* was induced (Supplementary Figure S2B). We found both protein and mRNA levels of *ompW* were decreased by the over-expression of *nlpI* in the deletion mutants of *ibpA*, *ibpB*, *ibpAB* and *ibpA* complementation strains (Supplementary Figures S2A,E). Interestingly, SDS-PAGE showed that OmpW was not decreased by increasing NlpI-M protein level (Figure 3F). These results indicated that the over-expression of full length *nlpI* inhibited the expression of *ompW* in an IbpA/B independent manner.

### C-terminus of *nlpI* involved in the transcription of *ibpA* and *ibpB*

**Figure 4:**
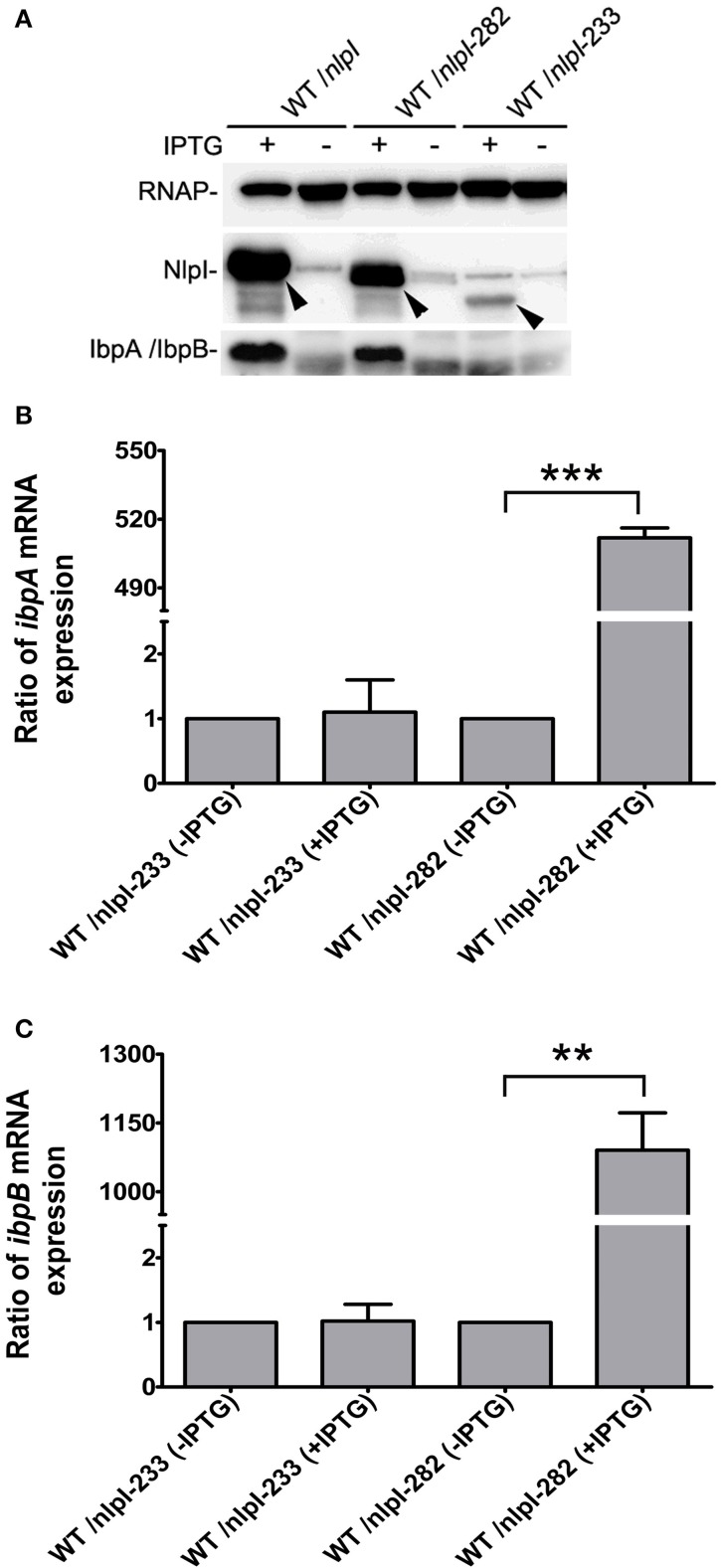
**C-terminal of NlpI involved in the transcription of *ibpA* and *ibpB*. (A)** Western blot with anti-NlpI and anti-IbpA antibody detected that IbpA and IbpB were increased in CP after the induction of *nlpI* or *nlpI*-282, but not in the over-expression of *nlpI*-233 cells. **(B, C)** qRT-PCR showed that the mRNA levels of *ibpA* and *ibpB* after the induction of *nlpI*-282 were increased by 511.8 ± 4.4, 1090.7 ± 81.4 fold, respectively, compared with corresponding stain without IPTG. But the mRNA levels of *ibpA* and *ibpB* after the induction of *nlpI*-233 were 1.1 ± 0.5, 1.02 ± 0.26 fold, respectively, compared with corresponding stain without IPTG. ^***^P < 0.001, ^**^P < 0.01.

It has been shown that the C-terminus of NlpI is critical to its function (Tadokoro et al., 2004), we want to test the role of the C-terminal NlpI in the transcription of *ibpA* and *ibpB* by over-expressing of *nlpI-*282 or *nlpI-*233 in the wild type strain. Western blot showed that the amount of IbpA/IbpB was increased in CP fraction in the over-expression of *nlpI-*282 cells, but not in the over-expression of *nlpI-*233 cells (Figure 4A). qRT-PCR showed that the levels of *ibpA* and *ibpB* mRNA were not elevated in the over-expression of *nlpI-*233 cell, but increased by 511.8 ± 4.4 and 1090.7 ± 81.4 fold after the induction of *nlpI-*282, respectively (Figures 4B,C, Supplementary Figure S3). These results indicated that C-terminus of NlpI played an important role in the transcription of *ibpA* or *ibpB*.

### *nlpI* mediated IbpA and IbpB localization to outer membrane

**Figure 5:**
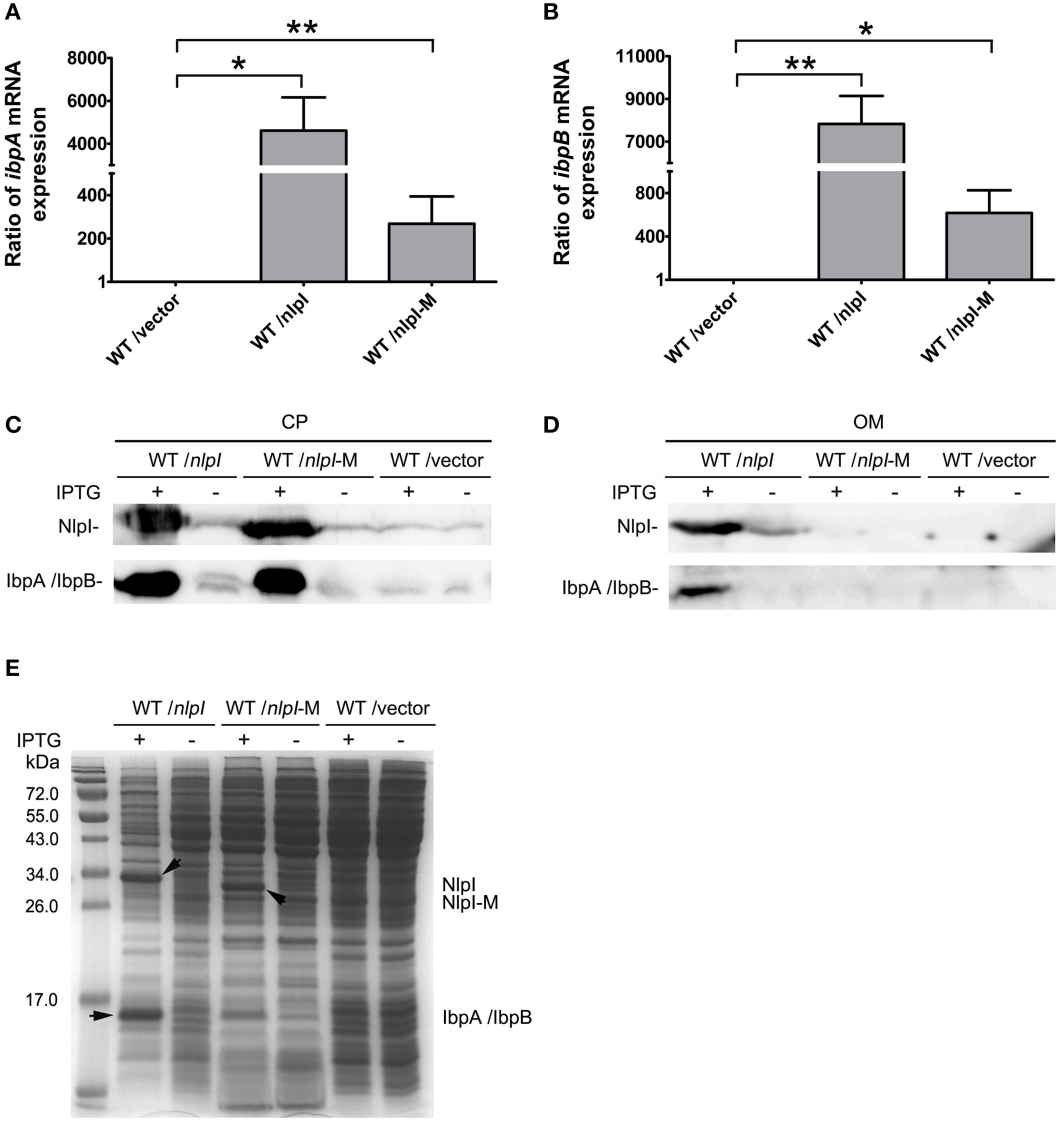
**NlpI mediated IbpA and IbpB localization to outer membrane**. **(A, B)** qRT-PCR showed that the mRNA levels of *ibpA* **(A)** and *ibpB* **(B)** by over-expression of *nlpI* or *nlpI*-M were increased dramatically, compared with control. **(C)** Western blot probed with anti-NlpI and anti-IbpA antibody showed that over-expression of *nlpI* or *nlpI*-M induced IbpA and/or IbpB in CP. **(D)** Western blot with anti-HisTag and anti-IbpA antibody showed that NlpI and IbpA/IbpB were present in OM of MG1655/pQE80-*nlpI* with inducer, but did not detected NlpI and IbpA/IbpB in OM of MG1655/pQE80-*nlpI*-M with inducer. **(E)** The CP protein profiling of MG1655/pQE80-*nlpI*, MG1655/pQE80-*nlpI*-M and MG1655/pQE80L with/without inducer. ^**^P < 0.01, ^*^P < 0.05.

It has been shown that IbpA and IbpB are localized to OM (Laskowska et al., 1996), and our data showed that over-expression of *nlpI* promoted the expression of *ibpA* and *ibpB*, we speculate that the localization of IbpA and IbpB might be affected. To test this hypothesis, we over-expressed *nlpI* or *nlpI*-M in the wild type cells followed by RNA isolation and cell fractionation. qRT-PCR showed that the mRNA levels of *ibpA* and *ibpB* were increased after the over-expression of *nlpI* or *nlpI*-M (Figures 5A,B). We detected the dramatic increase of NlpI in the OM of cells with over-expression of *nlpI* by using His-tag antibody, but not in the cells with over-expression of *nlpI*-M (Figure 5D). This result showed that N-terminal signal sequence is required for NlpI localization to OM. The amount of IbpA/IbpB was increased in CP fraction after the over-expression of either *nlpI* or *nlpI*-M as expected (Figures 5C,E). However, the amount of IbpA/IbpB in OM fraction was increased only after the over-expression of *nlpI*, but not *nlpI-*M (Figure 5D). These data indicated that localization of NlpI to OM is related to the process of IbpA and IbpB localizing to OM.

### NlpI physically interacts with IbpB

**Figure 6:**
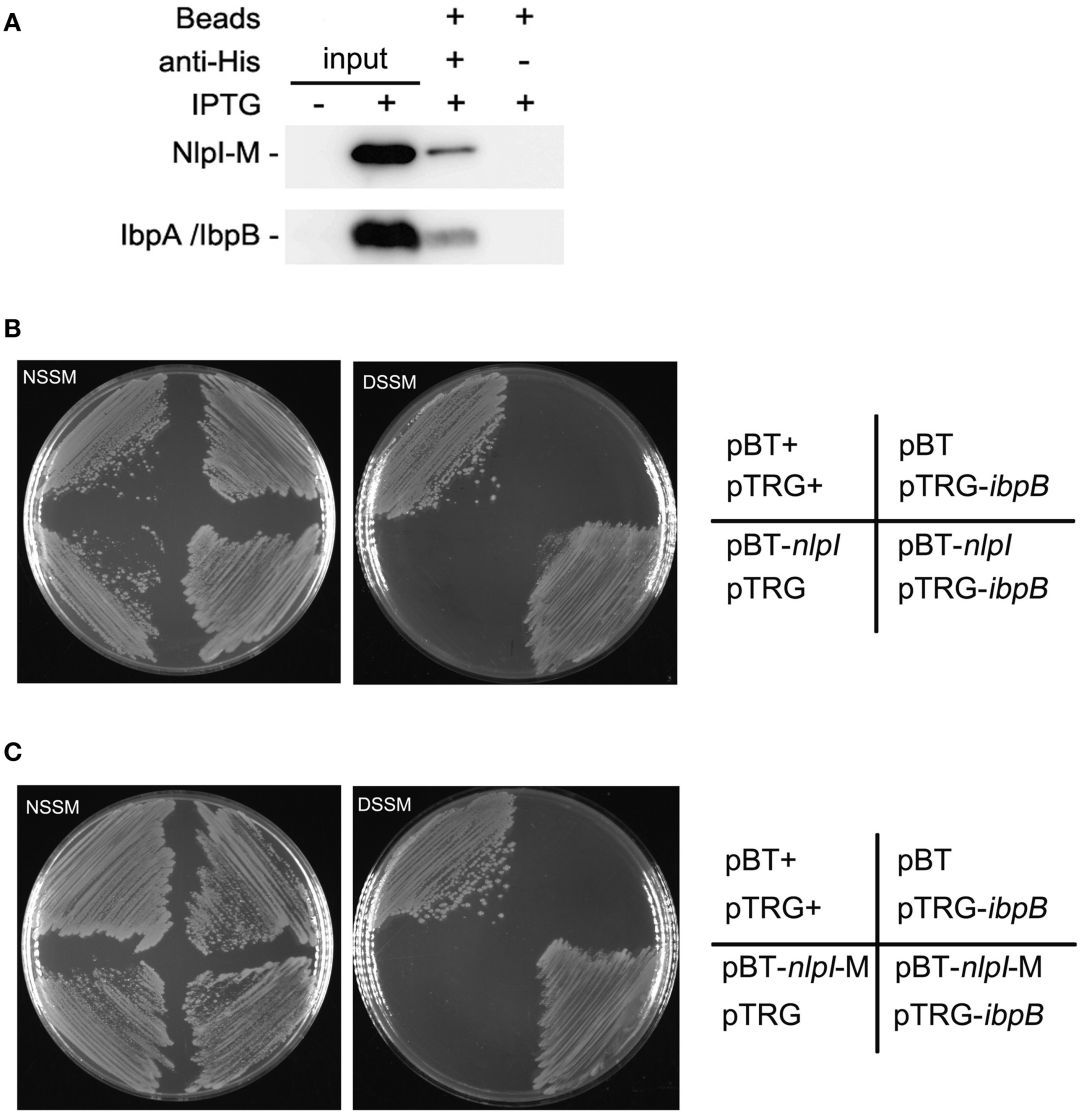
**NlpI physically interacts with IbpB. (A)** Co-IP assays for the binding of NlpI-M with protein A beads by using anti-HisTag antibody. Immune complexes in MG1655/pQE80-*nlpI*-M induced with IPTG were collected, and the beads were washed with IP Lysis/Wash buffer. The samples were analyzed by using anti-IbpA antibody and anti-HisTag antibody, respectively. Lane 1 and lane 2 represented input CP of the bacteria without or with IPTG for Co-IP assay. Lane 3 and lane 4 are results of Co-IP assay. **(B, C)** Bacterial two-hybrid assays detected the interaction between IbpB and NlpI **(B)** or NlpI-M **(C)**. NSSM, Non-selective Screening Medium. DSSM, Dual Selective Screening Media (plate contained str and 5 mM 3-AT). pBT^+^ and pTRG^+^, co-transformant containing pBT-LGF2 and pTRG-Gal11P as a positive control.

Upregulation of IbpA and IbpB by NlpI over-expression and their co-localization to the OM suggested that these two proteins may function together with NlpI to cause growth retardation. Thus, we checked the interaction between NlpI and IbpA or IbpB. Co-immunoprecipitation (Co-IP) assay was applied to detect the potential interaction between NlpI and IbpA/IbpB. Co-IP assay was performed by binding of His-tag antibody to protein A/G plus agarose beads and incubating with cell extracts. Immune complexes were eluted in elution buffer and analyzed by Western blot using anti-IbpA and anti-HisTag antibody (Figure 6A). The results showed that NlpI interacted with IbpA and/or IbpB. We expressed and purified NlpI-M in wild type strain, Δ*ibpA*, Δ*ibpB* or Δ*ibpAB*, respectively. Western blot and MS analysis showed IbpA and IbpB were co-eluted when NlpI-M was purified in wild type strain (Supplementary Figure S4 and Table S5). Moreover, Western blot confirmed that IbpB was co-eluted in purified NlpI-M from Δ*ibpA*. We used another method, bacterial two-hybrid assay to confirm the interaction between NlpI and IbpA/IbpB. As shown in Figures 6B,C, the co-transformants containing IbpB and NlpI or NlpI-M grew well-on DSSM. But the co-transformants containing IbpA and NlpI or NlpI-M had no obvious growth phenomenon on DSSM (data no shown). These results suggested that NlpI, IbpA, and IbpB may form a complex to mediate the cell division.

## Discussion

In this study, we found that the over-expression of NlpI severely inhibited the bacterial growth, influenced nucleoids division and FtsZ localization in the septum, and that *nlpI*-282 lacking C-terminal 12 amino acid residues sequence showed similar phenotypes. However, the over-expression of *nlpI*-M and *nlpI*-233 failed to inhibit nucleoids division and FtsZ localization. Considering the fact that we did not detect the interaction between NlpI and FtsZ, we speculated that NlpI participated in cell division by inhibiting nucleoid division and interfering with FtsZ localization in a contact independent manner.

The previous study showed that the over-expression of recombinant proteins result in heat shock-like response in *E. coli* (Gill et al., 2000). Under this condition, the mRNA levels of *ibpA* and *ibpB* are highly expressed (Gill et al., 2000; Jurgen et al., 2000). IbpA and IbpB are molecular chaperone proteins (Jiao et al., 2005; Strozecka et al., 2012), which are involved in resistance to heat and superoxide stresses (Kitagawa et al., 2000) and protect enzymes from inactivation by heat (Kitagawa et al., 2002). IbpA decreases the size of substrate complexes and inhibits their further processing (Ratajczak et al., 2009). IbpB, which is associated with its substrate via forming complexes with IbpA (Matuszewska et al., 2005), facilitates substrate transfer to the Hsp70/40 and the Hsp100 chaperone machinery (Ratajczak et al., 2009). In this study, the DNA microarray analysis showed that the expression of IbpA, IbpB, the Hsp70 chaperone DnaK and the Hsp100 chaperone ClpB significantly increased after the over-expression of *nlpI* (Table 2). Furthermore, the protein amount of IbpA and IbpB were significantly increased in CP and OM. The deletion of *ibpA* or *ibpB* and the trans-complementation of Δ*ibpA* experiments indicated that both IbpA and IbpB were involved in the NlpI-participated cell division.

Although the increase of NlpI protein level induced the expression of *ibpA* and *ibpB* and disrupted the bacterial nucleoids division, over-expression of *ibpA* and/or *ibpB* did not show similar phenotypes. In Δ*ibpA*, Δ*ibpB* or Δ*ibpAB*, nucleoids division was normal under the over-expression of *nlpI*, but the nucleoids were anomalous in the trans-complementation strains of Δ*ibpA* with the over-expression of *nlpI* (Supplementary Figure S2D). The results indicated that inhibition nucleoids division of NlpI was dependent on the heat shock proteins IbpA and IbpB. Over-expression of *nlpI*-M did not inhibit the bacterial growth and nucleoid division, but induced the mRNA levels of *ibpA* and *ibpB*. Moreover, the over-expression of *nlpI*-M caused the increase of IbpA/IbpB in CP, but not in OM. These results demonstrated that localization of IbpA/IbpB in OM played an important role in the NlpI-participated cell division. Co-immunoprecipitation assay, proteins MS analysis (Supplementary Figure S4 and Table S5) and bacterial two-hybrid assay showed that NlpI physically interacts with IbpB. Moreover, IbpA was not found to interact with NlpI, which highly suggests that the interaction between IbpB and NlpI is specific. It has been shown that IbpA interacts with IbpB (Matuszewska et al., 2005). We speculated that localization of NlpI in OM was essential for the export of IbpB, together with IbpA to OM and required for the NlpI-participated cell division. IbpA and IbpB play important roles in protecting recombinant proteins from degradation by cytoplasmic proteases (Han et al., 2004) and optimize recombinant proteins de novo folding (de Marco et al., 2007). Here, we found that IbpAB are esstential mediators in NlpI-participated cell division process. This is different from their above mentioned chaperones roles. We propose that IbpA, IbpB and NlpI form a protein complex, which plays an essential role in the detrimental effect of NlpI over-expression because deletion of IbpA or IbpB abolished the effect. This unkown protein complex will be explored in the future to find out the role of IbpAB in the NlpI-participated cell division.

The previous studies reported that the insertion mutant of *nlpI* shows osmotic-sensitive growth at 42°C in *E. coli* (Ohara et al., 1999), and *nlpI* is up-regulated in response to high-pressure and the deletion mutant of *nlpI* is more sensitive to high-pressure than the wild type strain (Malone et al., 2006; Charoenwong et al., 2011). In this study, we found that the growth of Δ*nlpI* was inhibited more severely than the wild type strain at 50°C (Figure S5). Moreover, NlpI contributes to the cold acclimatization response in *Salmonella* Thyphimurium (Rouf et al., 2011b). We speculate that NlpI is a stress response protein and especially responds to high temperature. The up-regulation of *ibpA* and *ibpB* may be linked to the NlpI-participated stress response.

In summary, we found that over-expression of *nlpI* interrupts the nucleoids division and the assembly of FtsZ at the septum, and IbpA/B are required for this process, possibly by forming a NlpI/IbpA/IbpB protein complex. Other unknown factor(s) must be involved in the cell division defect caused by over-expression of *nlpI*. Since NlpI is a potential stress response protein, we proposed that NlpI can slow down the cell growth by inhibiting cell division to contribute the host survival when bacteria encounter heat shock stress.

### Conflict of interest statement

The Review Editor Ching-Hao Teng declares that, despite having collaborated with author Yufeng Yao, the review process was handled objectively and no conflict of interest exists. The authors declare that the research was conducted in the absence of any commercial or financial relationships that could be construed as a potential conflict of interest.
