# Supplementary material for: Heat shock proteins IbpA and IbpB are required for NlpI-participated cell division in Escherichia coli
Source: Front Microbiol. 2015 Feb 4;6:51. doi: 10.3389/fmicb.2015.00051 (PMC4316790; doi:10.3389/fmicb.2015.00051)
Supplement: Supplementary file 1 [file DataSheet1.PDF]

Supplementary Material

Heat shock proteins IbpA and IbpB are required for NlpI-participated cell division in *Escherichia coli*

Jing Tao[1], Yu Sang[1], Qihui Teng[1], Jinjing Ni[1], Yi Yang[2], Stephen Tsui[2], Yu-Feng Yao[1]*

[1] Laboratory of Bacterial Pathogenesis, Department of Microbiology and Immunology, Institutes of Medical Sciences, Shanghai Jiao Tong University School of Medicine, Shanghai, China 200025

[2] School of Biomedical Sciences, The Chinese University of Hong Kong, Hong Kong SAR, China

***Correspondence:** Dr. Yu-Feng Yao, Department of Microbiology and Immunology, Institutes of Medical Sciences, Shanghai Jiao Tong University School of Medicine, Shanghai 200025, China

yfyao@sjtu.edu.cn

Supplementary Tables and Figures

Supplementary Tables

Table S1. Primers used in the current study

Primers	Sequence	Notes
nlpI KO F	ATGAAGCCTTTTTTGCGCTGGTGTTTCGTTGCGACAGCAC TTACGCTTGCCATATGAATATCCTCCTTAG	*nlpI* knock out
nlpI KO R	CTATTGCTGGTCCGATTCTGCCAGGTCATCTTGGTCCTGG CCCAGGAGCGGTGTAGGCTGGAGCTGCTTC	*nlpI* knock out
ibpAB KO F	GTCCATTGTGGAAGGTCTTACATTCTCGCTGATTTCAGGA GCTATTGATTGTGTAGGCTGGAGCTGCTTC	*ibpAB* knock out
ibpAB KO R	GTAAGACAAAAAAAGCCCCACCAGAATGGCGGGGCAAA GAGAATAGCTAGCATATGAATATCCTCCTTA	*ibpAB* knock out
ibpB KO F	AATCTTCGGATTTGCAGGTACTTACTCGCTTCTTAGAAGG AGAAATGACTGTGTAGGCTGGAGCTGCTTC	*ibpB* knock out
ibpA KO R	CCTGACGGCGAGCATGGAGATGTCAGGCCGCGCCAGGC GGCCTTAGGGAACATATGAATATCCTCCTTA	*ibpA* knock out
ompW KO F	CAATGTAGGTATATTCGTCACGTTTTTATAACCATAACGAC GGAGCGGATGTGTAGGCTGGAGCTGCTTC	*ompW* knock out
ompW KO R	TACCATGTCCTTATTGACCCCGTATATTACGGGGTCGTTTT TGTGCGGAACATATGAATATCCTCCTTA	*ompW* knock out
nlpI-AfeI F	ATGAAGCCTTTTTTGCGCTG	pQE80-*nlpI* construction
nlpI-FseI R	TATAGGCCGGCCTTTGCTGGTCCGATTCTGCCAGG	pQE80-*nlpI* construction
nlpI-M-AfeI F	ATGAGTAATACTTCCTGGCGTAAAAG	pQE80-*nlpI*-M construction
nlpI-282-FseI R	TATAGGCCGGCCTGCCCAGGAGCGATAATTCCAAC	pQE80-*nlpI*-282 construction
nlpI-233-FseI R	TATAGGCCGGCCTGAGATGCTCAGCGAGCGAGGTG	pQE80-*nlpI*-233 construction
ibpA-AfeI F	ATGCGTAACTTTGATTTATC	pQE80-*ibpA* construction
ibpA-FseI R	TATAGGCCGGCCTGTTGATTTCGATACGGCGCG	pQE80-*ibpA* construction
ibpB-AfeI F	ATGCGTAACTTCGATTTATC	pQE80-*ibpB* construction
ibpB-FseI R	TATAGGCCGGCCTGCTATTTAACGCGGGACGTT	pQE80-*ibpB* construction
ibpA EcoRV F	TTCCGCTGCATATCATGAAC	pACYC184-*ibpA* promoter construction

ibpA BamHI R	CGGGATCCTTAGTTGATTTCGATACGGC	pACYC184-*ibpA* promoter construction
nlpI-EcoRI F	CGGAATTCCATGAAGCCTTTTTTGCGCTG	pBT-*nlpI* construction
nlpI-XhoI R	CCGCTCGAGTTGCTGGTCCGATTCTGCCA	pBT-*nlpI* construction
nlpI-M-EcoRI F	CGGAATTCCATGAGTAATACTTCCTGGCG	pBT-*nlpI*-M construction
nlpI-BamHI-F	CGGGATCCATGAAGCCTTTTTTGCGCTG	pTRG-*nlpI* construction
nlpI-M-BamHI F	CGGGATCCATGAGTAATACTTCCTGGCG	pTRG-*nlpI*-M construction
ibpA-BamHI F	CGGGATCCATGCGTAACTTTGATTTATC	pTRG-*ibpA* construction
ibpA-XhoI R	CCGCTCGAGGTTGATTTCGATACGGCGCG	pTRG-*ibpA* construction
ibpB-BamHI F	CGGGATCCATGCGTAACTTCGATTTATC	pTRG-*ibpB* construction
ibpB-XhoI R	CCGCTCGAGGCTATTTAACGCGGGACGTT	pTRG-*ibpB* construction
ibpA-EcoRI F	CGGAATTCCATGCGTAACTTTGATTTATC	pBT-*ibpA* construction
ibpB-EcoRI F	CGGAATTCCATGCGTAACTTCGATTTATC	pBT-*ibpB* construction
nlpI-F	GTCTCGGTCTGAGGGCATTA	qRT-PCR
nlpI-R	TGCGCGTAGTTGTAAGTTGG	qRT-PCR
ibpA-F	CTATCGCTGTGGCTGGTTTT	qRT-PCR
ibpA-R	ACCAGGTTAGCACCACGAAC	qRT-PCR
ibpB-F	GGAGCAGCCAAAAGAAGAGA	qRT-PCR
ibpB-R	ATGGGTTCAGGCTCATTACG	qRT-PCR
ompW-F	TTCTCTCTGGCAGTGCCTTT	qRT-PCR
ompW-R	TGCCAGTAATTCCACACCAA	qRT-PCR
16S rRNA-F	CAGCCACACTGGAACTGAGA	qRT-PCR
16S rRNA-R	GTGCTTCTTCTGCGGGTAAC	qRT-PCR

Table S2. Genes upregulated by over-expression of *nlpI*

Annotation	Gene	LogFC	P.Val
heat shock protein IbpA	*ibpA*	7.25	1.57E-06
lipoprotein NlpI	*nlpI*	7.23	1.57E-06
heat shock chaperone IbpB	*ibpB*	7.04	2.54E-06
tryptophanase leader peptide	*tnaL*	5.80	5.32E-06
dihydrolipoamide dehydrogenase	*lpdA*	4.00	9.47E-04
co-chaperonin GroES	*groES*	3.96	2.13E-04
50S ribosomal protein L5	*rplE*	3.88	4.38E-04
heat shock protein 90	*htpG*	3.69	2.48E-04
chaperonin GroEL	*groEL*	3.68	5.07E-03
lac repressor	*lacI*	3.50	3.79E-04
cold shock protein CspE	*cspE*	3.44	7.05E-03
D-ribose transporter subunit RbsB	*rbsB*	3.23	2.61E-03
mannonate dehydratase	*uxuA*	3.19	1.96E-03
fumarate/nitrate reduction transcriptional regulator	*fnr*	3.15	1.13E-03
DNA-binding transcriptional activator MarA	*marA*	3.05	1.23E-03
DNA-binding transcriptional repressor MarR	*marR*	2.91	2.39E-04
50S ribosomal protein L11	*rplK*	2.90	5.20E-04
outer membrane protein F	*ompF*	2.80	4.47E-03
elongation factor Tu	*tuf*	2.78	8.30E-03
protein disaggregation chaperone	*clpB*	2.57	2.58E-04
alpha-ketoglutarate transporter	*kgtP*	2.53	8.78E-04
oligopeptide ABC transporter	*oppA*	2.53	4.31E-03
hypothetical protein	*ybeD*	2.49	4.30E-03
hypothetical protein	*yeiC*	2.41	4.00E-04
mannonate dehydratase	*uxuA*	2.37	7.26E-04
molecular chaperone DnaK	*dnaK*	2.36	3.43E-03
galactitol-specific PTS system component IIB	*gatB*	2.35	8.67E-04
ATP-dependent protease ATP-binding subunit HslU	*hslU*	2.32	1.00E-03
cytochrome o ubiquinol oxidase subunit II	*cyoA*	2.29	4.00E-03
beta-D-glucuronidase	*uidA*	2.29	1.30E-03
mannonate oxidoreductase	*uxuB*	2.20	2.59E-03
trehalose(maltose)-specific PTS system	*treB*	2.14	1.06E-03
NADH dehydrogenase subunit B	*nuoB*	2.11	5.96E-04

DNA-binding ATP-dependent protease La	*lon*	2.10	1.12E-03
FxsA	*fxsA*	2.04	6.11E-04
calcium/sodium:proton antiporter	*chaA*	2.03	5.64E-04
aspartate ammonia-lyase	*aspA*	2.03	3.47E-03
universal stress protein, broad regulatory function?	*uspA*	2.03	2.14E-03
RNA polymerase sigma factor RpoS	*rpoS*	2.01	2.24E-03
outer membrane protein X	*ompX*	2.00	1.60E-03
biofilm formation regulatory protein BssS	*bssS*	1.94	6.54E-03
ATP-dependent Clp protease proteolytic subunit	*clpP*	1.89	6.44E-03
heat shock protein HtpX	*htpX*	1.88	3.95E-03
L-lactate permease	*lldP*	1.86	1.12E-03
transcriptional regulator HU subunit alpha	*hupA*	1.84	1.46E-03
glycerol-3-phosphate dehydrogenase	*glpD*	1.82	6.02E-03
hypothetical protein	*yccV*	1.81	1.84E-03
DNA mismatch repair protein	*mutL*	1.81	4.40E-03
isocitrate lyase	*aceA*	1.73	6.43E-03
succinate dehydrogenase flavoprotein subunit	*sdhA*	1.72	6.37E-03
type II secretion protein	*etpI*	1.69	1.35E-03
hypothetical protein	*yiiU*	1.69	4.91E-03
gamma-glutamyl-gamma-aminobutyrate hydrolase	*puuD*	1.69	4.27E-03
lipoprotein NlpD	*nlpD*	1.67	1.31E-03
succinylglutamate desuccinylase	*ydjS*	1.66	1.13E-03
chaperone protein DnaJ	*dnaJ*	1.66	2.19E-03
periplasmic repressor CpxP	*cpxP*	1.62	3.57E-03
hypothetical protein	*yohO*	1.54	3.93E-03
ATP-dependent protease peptidase subunit	*hslV*	1.44	2.02E-03
succinyl-CoA synthetase subunit alpha	*sucD*	1.41	3.61E-03
23S rRNA methyltransferase J	*rrmJ*	1.39	2.87E-03
hypothetical protein	*yijF*	1.34	2.33E-03
thiamine transporter membrane protein	*thiP*	1.27	3.28E-03
succinate dehydrogenase	*sdhD*	1.18	5.43E-03
thiamine monophosphate kinase	*thiL*	1.16	4.03E-03
formate hydrogenlyase, subunit E	*hycE*	1.15	5.45E-03
putative transport protein	*yaaU*	1.12	4.43E-03
hemolysin expression-modulating protein	*hha*	1.02	6.23E-03

LogFC: the log2-fold-change

Table S3. Genes downregulated by over-expression of *nlpI*

Annotation	Gene	LogFC	P.Val
anti-sigma 28 factor FlgM	*flgM*	-4.09	3.96E-03
outer membrane protein W	*ompW*	-1.91	9.83E-04
flagellar biosynthesis sigma factor	*fliA*	-1.86	4.78E-03
flagellar basal body rod modification protein	*flgD*	-1.84	1.92E-03
translation inhibitor protein RaiA	*yfiA*	-1.81	8.15E-04
outer membrane protein	*slp*	-1.80	8.73E-04
flagellar hook protein FlgE	*flgE*	-1.78	1.46E-03
flagellar basal body rod protein FlgG	*flgG*	-1.68	6.24E-03
cytochrome d terminal oxidase, polypeptide subunit I	*cydA*	-1.64	1.22E-03
cytochrome d terminal oxidase polypeptide subunit II	*cydB*	-1.62	1.66E-03
PTS system galactitol-specific enzyme IIC	*gatC*	-1.58	1.96E-03
flagellar basal body rod protein FlgB	*flgB*	-1.55	2.08E-03
flagellar motor switch protein FliN	*fliN*	-1.54	2.86E-03
glycine cleavage system protein H	*gcvH*	-1.54	6.23E-03
flagellar basal body rod protein FlgF	*flgF*	-1.54	1.54E-03
flagellar motor protein MotB	*motB*	-1.53	2.42E-03
maltose ABC transporter periplasmic protein	*malE*	-1.47	3.50E-03
hypothetical protein	*yidQ*	-1.44	5.86E-03
glycine dehydrogenase	*gcvP*	-1.44	3.44E-03
flagellar hook-associated protein FlgL	*flgL*	-1.41	2.07E-03
flagellar basal body P-ring biosynthesis protein FlgA	*flgA*	-1.39	4.53E-03
melibiose:sodium symporter	*melB*	-1.38	5.16E-03
L-asparaginase II	*ansB*	-1.36	2.64E-03
glutamine ABC transporter periplasmic protein	*glnH*	-1.32	4.36E-03
autonomous glycyl radical cofactor GrcA	*yfiD*	-1.29	4.83E-03
F0F1 ATP synthase subunit beta	*atpD*	-1.27	3.15E-03
flagellar basal body rod protein FlgC	*flgC*	-1.26	3.98E-03
30S ribosomal protein S12	*rpsL*	-1.25	3.40E-03
leucyl aminopeptidase	*pepA*	-1.24	5.94E-03
hypothetical protein	*yecA*	-1.24	7.58E-03
methyl-accepting chemotaxis protein III	*trg*	-1.23	7.49E-03
flagellar motor switch protein G	*fliG*	-1.23	5.95E-03
F0F1 ATP synthase subunit gamma	*atpG*	-1.22	3.58E-03

flagellar basal body L-ring protein	*flgH*	-1.20	5.37E-03
hypothetical protein	*ybeL*	-1.20	8.09E-03
glucose-1-phosphatase/inositol phosphatase	*agp*	-1.19	3.99E-03
thiol peroxidase	*tpx*	-1.18	5.64E-03
hypothetical protein	*yjaH*	-1.17	5.93E-03
phosphohistidinoprotein-hexose phosphotransferase	*ptsH*	-1.16	8.59E-03
flagellar capping protein	*fliD*	-1.16	4.26E-03
flagellar basal body-associated protein FliL	*fliL*	-1.15	4.78E-03
hypothetical protein	*yjcZ*	-1.14	4.25E-03
citrate reductase cytochrome c-type subunit	*napB*	-1.14	6.80E-03
anaerobic glycerol-3-phosphate dehydrogenase	*glpB*	-1.14	6.57E-03
chemotaxis regulatory protein CheY	*cheY*	-1.13	6.08E-03
hypothetical protein	*yqjE*	-1.13	4.87E-03
ammonium transporter	*amtB*	-1.13	9.61E-03
dihydrolipoamide acetyltransferase	*aceF*	-1.13	7.69E-03
glycogen synthesis protein GlgS	*glgS*	-1.12	5.47E-03
maltoporin	*lamB*	-1.12	5.85E-03
F0F1 ATP synthase subunit epsilon	*atpC*	-1.11	5.62E-03
primosomal replication protein N	*priB*	-1.11	9.98E-03
maltose transporter permease	*malG*	-1.11	5.05E-03
cysteinyl-tRNA synthetase	*cysS*	-1.09	7.89E-03
acetyl-CoA carboxylase carboxyltransferase	*accA*	-1.09	5.46E-03
isocitrate dehydrogenase	*icdA*	-1.09	7.87E-03
serine hydroxymethyltransferase	*glyA*	-1.09	7.73E-03
proline aminopeptidase P II	*pepP*	-1.08	6.62E-03
chemotaxis methyltransferase CheR	*cheR*	-1.08	4.90E-03
flagellar rod assembly protein/muramidase FlgJ	*flgJ*	-1.07	5.18E-03
fumarate reductase flavoprotein subunit	*frdA*	-1.07	7.34E-03
F0F1 ATP synthase subunit C	*atpE*	-1.07	5.28E-03
anaerobic dimethyl sulfoxide reductase subunit B	*dmsB*	-1.06	7.99E-03
chemotaxis regulator CheZ	*cheZ*	-1.06	8.80E-03
hypothetical protein	*ychK*	-1.06	6.76E-03
hypothetical protein	*yihD*	-1.05	8.14E-03
DNA starvation/stationary phase protection protein	*dps*	-1.05	7.16E-03
undecaprenyldiphospho-muramoylpentapeptide beta-N-acetylglucosaminyltransferase	*murG*	-1.05	5.45E-03

flagella synthesis protein FlgN	*flgN*	-1.04	5.45E-03
flagellar basal body P-ring protein	*flgI*	-1.04	6.02E-03
pyruvate dehydrogenase subunit E1	*aceE*	-1.03	7.61E-03
peptide chain release factor-like protein	*prfH*	-1.02	9.49E-03
sugar fermentation stimulation protein A	*sfsA*	-1.02	7.88E-03
maltose/maltodextrin transporter ATP-binding protein	*malK*	-1.01	9.72E-03

LogFC: the log2-fold-change

Table S4. Peptides identified by MS

Identified protein	Sequence positions	Sequence
IbpA	1 – 13	MIMRNFDLSPLYR
	14 – 20	SAIGFDR
	21 – 50	LFNHLENNQSQSNGGYPPYNVELVDENHYR
	51 – 75	IAIAVAGFAESELEITAQDNLLVVK
	76 – 85	GAH ADEQKER
	86 – 95	TYLYQGIAER
	100 – 110	KFQLAENIHVR
	111 – 125	GANLVNGLLYIDLER
OmpW	1 – 9	HEAGEFFMR
	69 – 95	ATGDIATVHHLPPTLMAQWYFGDASSK
	96 – 119	FRPYVGAGINYTTFFDNGFNDHGK
	120 – 129	EAGLSDLSLK
	130 – 146	DSWGAAGQVGVDYLINR
	147 – 166	DWLVNMSVWYMDIDTTANYK
	167 – 190	LGGAQQHDSVRLDPWVFMFSAGYR

Table S5. Peptides identified by MS

Identified protein	Sequence positions	Sequence
IbpA	1 – 11	MRNFDLSPLYR
	21 – 50	LFNHLENNQSQSNGGYPPYNVELVDENHYR
	76 – 85	GAH ADEQKER
	86 – 95	TYLYQGIAER
	100 – 110	KFQLAENIHVR
	111 – 131	GANLVNGLLYIDLERVIPEAK
IbpB	1 – 11	MRNFDLSPLMR
	18 – 39	LANALQNAGESQSFPPYNIEK
	47 – 59	ITLALAGFRQEDL
	59 – 71	LEIQLEGTRLSVK
	72 – 81	GTPEQPKEEK
	122 – 131	NEPEPIAAQR
	132 – 142	IAISERPALNS

Supplementary Figures

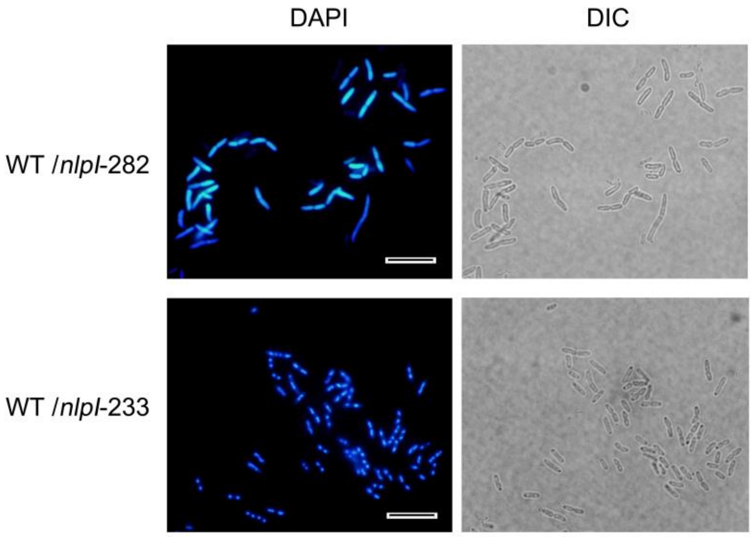

Figure S1. *E. coli* strain MG1655 with the plasmid pQE80-*nlpI*-233 or pQE80-*nlpI*-282 in the presence of 0.5 mM IPTG was stained with DAPI and nucleoids were observed by fluorescence microscope. Magnification, X1,000. Bar, 10 μm.

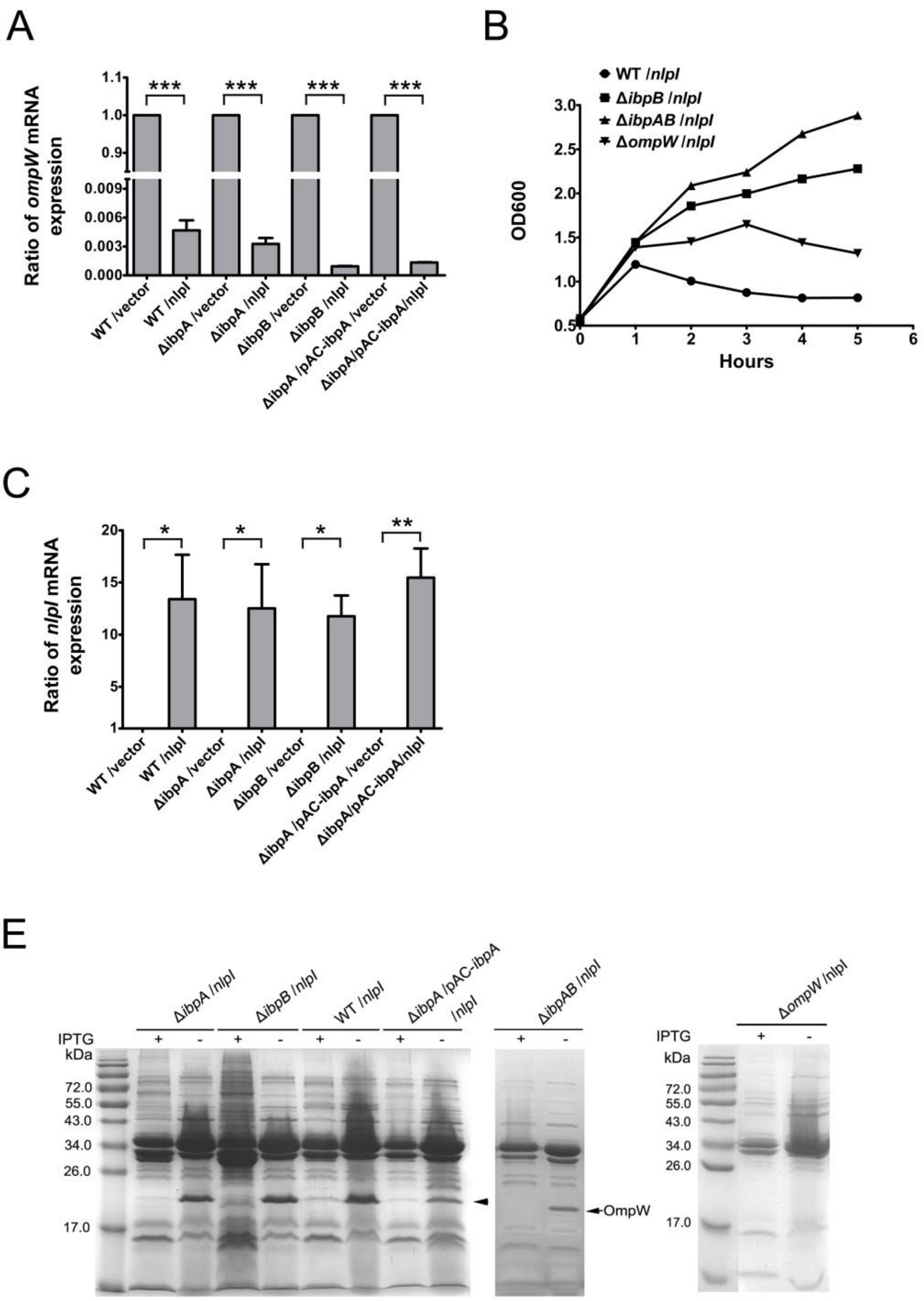
A
Ratio of ompW mRNA expression
1.1
1.0
0.9
0.009
0.006
0.003
0.000
***
WT /vector
WT /nlpI
ΔibpA /vector
ΔibpA /nlpI
ΔibpB /vector
ΔibpB /nlpI
ΔibpA /pAC-ibpA /vector
ΔibpA/pAC-ibpA/nlpI
B
WT /nlpI
ΔibpB /nlpI
ΔibpAB /nlpI
ΔompW /nlpI
OD600
Hours
C
Ratio of nlpI mRNA expression
20
15
10
5
1
*
**
E
ΔibpA /nlpI
ΔibpB /nlpI
WT /nlpI
ΔibpA /pAC-ibpA /nlpI
ΔibpAB /nlpI
ΔompW /nlpI
IPTG
kDa
72.0
55.0
43.0
34.0
26.0
17.0
OmpW

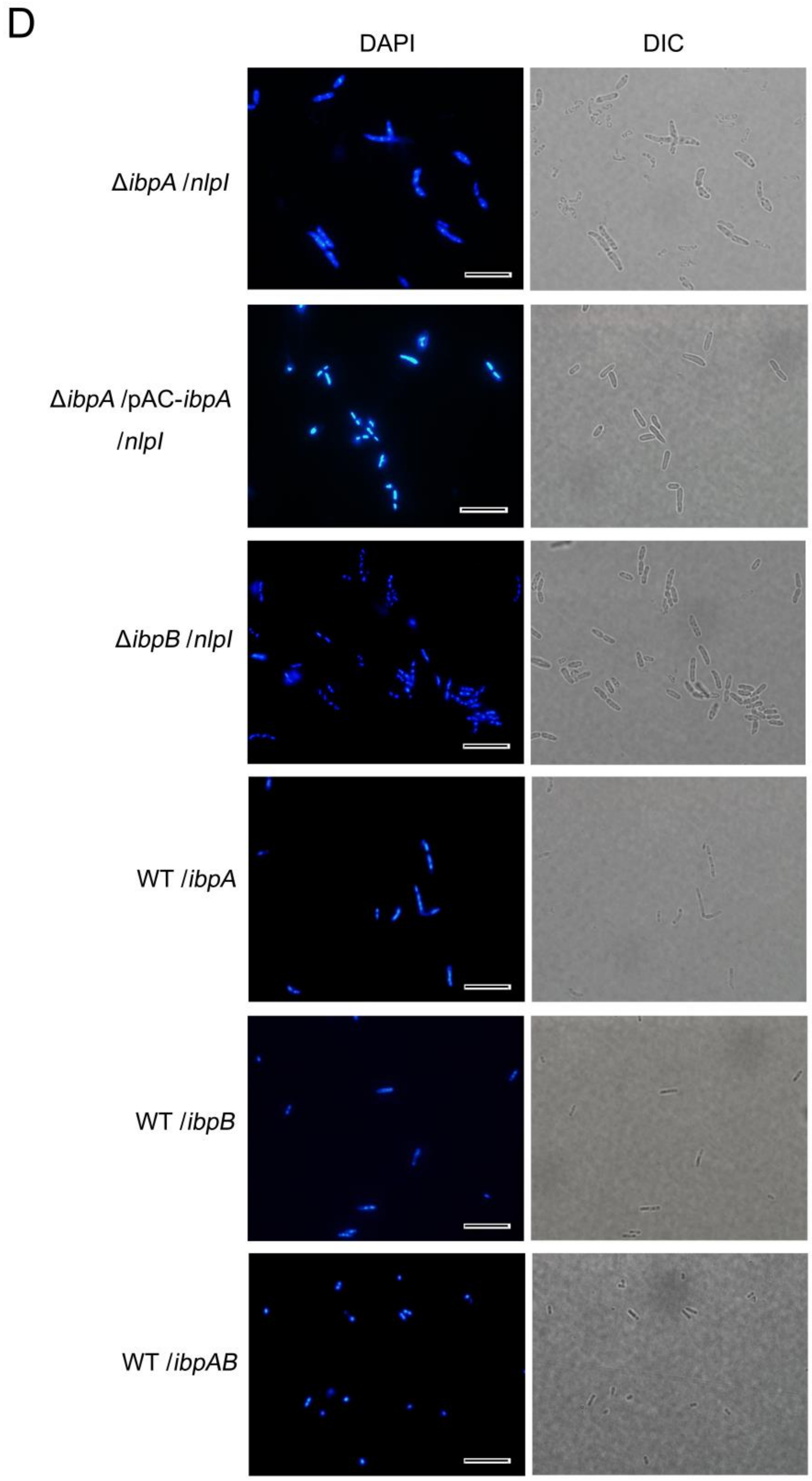

Figure S2. (A) qRT-PCR detected the level of *ompW* mRNA in different stains. The levels of *ompW* mRNA of different stains without IPTG were set at 1, respectively. The

level of *ompW* mRNA of WT, Δ*ibpA*, Δ*ibpB* or Δ*ibpA*/pAC-*ibpA* with IPTG induction was 0.0047 ± 0.0011, 0.0033 ± 0.0006, 0.0009 ± 0.00004, 0.0013 ± 0.00003 fold (means ± SD), respectively, compared with corresponding stain without IPTG. (B)The growth curves of *E. coli* strain MG1655, Δ*ibpB*, Δ*ibpAB* or Δ*ompW* with pQE80-*nlpI* in the presence of 0.5 mM IPTG were determined as described in the Materials and Methods. Three individual experiments were carried out. (C) qRT-PCR detected the level of *nlpI* mRNA in different stains. The levels of *nlpI* mRNA in different stains without IPTG were set at 1, respectively. The level of *nlpI* mRNA of WT, Δ*ibpA*, Δ*ibpB* or Δ*ibpA*/pAC-*ibpA* with IPTG induction increased by 13.4 ± 4.3, 12.5 ± 4.2, 11.8 ± 2.0, 15.5 ± 2.8 fold (means ± SD), respectively. The error bars indicated the SD representing the means from three independent experiments. (D) Δ*ibpA*/pQE80-*nlpI*, Δ*ibpA*/pAC-*ibpA*/pQE80-*nlpI*, Δ*ibpB*/pQE80-*nlpI*, MG1655/pQE80-*ibpA*, MG1655/pQE80-*ibpB* or MG1655/pQE80-*ibpAB* in the presence of 0.5 mM IPTG was stained with DAPI, and nucleoids were observed by fluorescence microscope. Magnification, X 1,000. Bar, 10 μm. (E) SDS-PAGE showed that the over-producing of *nlpI* in wild type strain, Δ*ibpA*, Δ*ibpB*, Δ*ibpAB* or Δ*ibpA*/pAC-*ibpA* decreased the expression of OmpW in OM. *** P<0.001, ** P<0.01, * P<0.05.

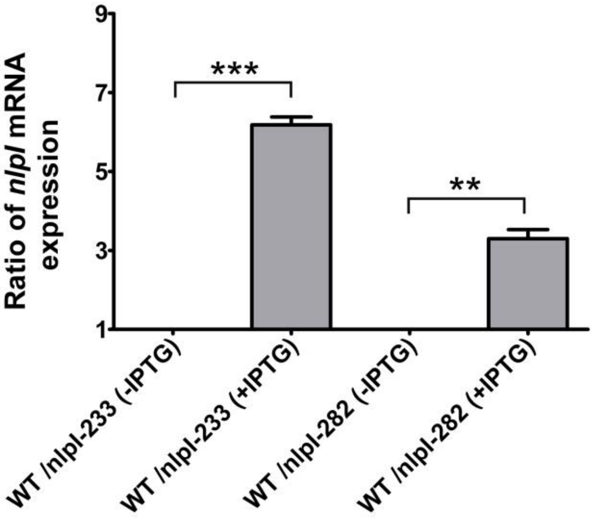

Figure S3. qRT-PCR showed that the mRNA levels of *nlpI* after the induction of *nlpI*-233 and *nlpI*-282 were increased by 6.18 ± 0.20, 3.30 ± 0.23 fold, respectively, compared with corresponding stain without IPTG. *** P<0.001, ** P<0.01.

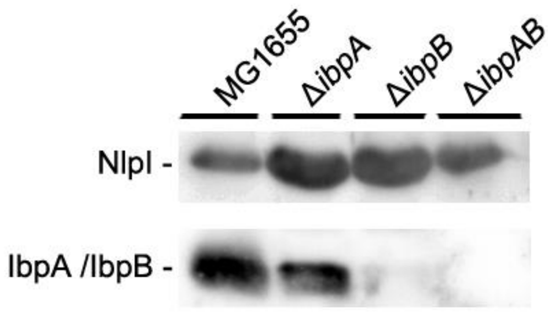

Figure S4. Protein purified assay suggested that NlpI-M interacted with IbpB. The wild type strain and its derivative mutants Δ*ibpA*, Δ*ibpB* or Δ*ibpAB* were transformed with the recombinant plasmid pQE80-*nlpI*-M, respectively. These strains were induced with 0.5 mM IPTG for 4 h followed by nickel affinity chromatography. Western blot tested the purified NlpI-M by anti-NlpI and anti-IbpA antibodies. The results showed that IbpB was co-eluted with NlpI-M from Δ*ibpA*. IbpA and /or IbpB were co-eluted with NlpI-M from wild type strain. MS confirmed that IbpA and IbpB co-purified with NlpI-M (Table S5).

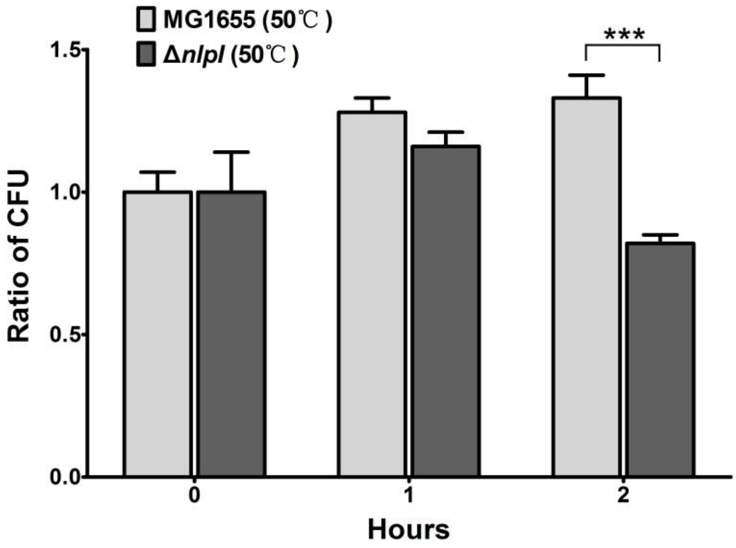

Figure S5. The growth of *E. coli* strain MG1655 and Δ*nlpI* at 50ºC. Overnight cultures of MG1655 and Δ*nlpI* were subcultured in 20 ml LB broth (1:100) and incubated at 30ºC with agitation until the OD600 was 0.4. The 0.1 ml cultures of different strains were diluted, coated plates and counted colonies as the zero hour. Then the residual cultures were continued to incubate in 50ºC while shaking at 250 rpm for 2 h. The CFU in different strains at the zero hour were set at 1, respectively. The CFU of wild type strain and Δ*nlpI* at 2 h were 1.33 ± 0.08, 0.82 ± 0.03 fold (means ± SD), respectively, compared with corresponding stains at the zero hour. *** P<0.001.
